# Supplementary material for: Comorbidity of mental ill-health in tuberculosis patients under treatment in a rural province of South Africa: a cross-sectional survey
Source: BMJ Open. 2022 Nov 21;12(11):e058013. doi: 10.1136/bmjopen-2021-058013 (PMC9680183; doi:10.1136/bmjopen-2021-058013)
Supplement: Author's manuscript [file bmjopen-2021-058013.draft_revisions.pdf]

BMJ Open

BMJ Open is committed to open peer review. As part of this commitment we make the peer review history of every article we publish publicly available.

When an article is published we post the peer reviewers' comments and the authors' responses online. We also post the versions of the paper that were used during peer review. These are the versions that the peer review comments apply to.

The versions of the paper that follow are the versions that were submitted during the peer review process. They are not the versions of record or the final published versions. They should not be cited or distributed as the published version of this manuscript.

BMJ Open is an open access journal and the full, final, typeset and author-corrected version of record of the manuscript is available on our site with no access controls, subscription charges or pay-per-view fees (http://bmjopen.bmj.com).

If you have any questions on BMJ Open's open peer review process please email info.bmjopen@bmj.com

BMJ Open

Prevalence of Co-Morbid Mental Ill-Health in Tuberculosis Patients under Treatment in a Rural Province of South Africa.

Journal:	*BMJ Open*
Manuscript ID	bmjopen-2021-058013
Article Type:	Original research
Date Submitted by the Author:	05-Oct-2021
Complete List of Authors:	Thungana, Yanga; Walter Sisulu University, Department of Psychiatry and Behavioural Sciences<br>Wilkinson, Robert; Francis Crick Institute, Pathology; Imperial College London<br>Zingela, Zukiswa; Nelson Mandela University, Executive Dean's Office, Faculty of Health Sciences
Keywords:	Tuberculosis < INFECTIOUS DISEASES, Substance misuse < PSYCHIATRY, Adult psychiatry < PSYCHIATRY

SCHOLARONE™
Manuscripts

I, the Submitting Author has the right to grant and does grant on behalf of all authors of the Work (as defined in the below author licence), an exclusive licence and/or a non-exclusive licence for contributions from authors who are: i) UK Crown employees; ii) where BMJ has agreed a CC-BY licence shall apply, and/or iii) in accordance with the terms applicable for US Federal Government officers or employees acting as part of their official duties; on a worldwide, perpetual, irrevocable, royalty-free basis to BMJ Publishing Group Ltd ("BMJ") its licensees and where the relevant Journal is co-owned by BMJ to the co-owners of the Journal, to publish the Work in this journal and any other BMJ products and to exploit all rights, as set out in our licence.

The Submitting Author accepts and understands that any supply made under these terms is made by BMJ to the Submitting Author unless you are acting as an employee on behalf of your employer or a postgraduate student of an affiliated institution which is paying any applicable article publishing charge ("APC") for Open Access articles. Where the Submitting Author wishes to make the Work available on an Open Access basis (and intends to pay the relevant APC), the terms of reuse of such Open Access shall be governed by a Creative Commons licence – details of these licences and which Creative Commons licence will apply to this Work are set out in our licence referred to above.

Other than as permitted in any relevant BMJ Author's Self Archiving Policies, I confirm this Work has not been accepted for publication elsewhere, is not being considered for publication elsewhere and does not duplicate material already published. I confirm all authors consent to publication of this Work and authorise the granting of this licence.

For peer review only

Prevalence of Co-Morbid Mental Ill-Health in Tuberculosis Patients under Treatment in a Rural Province of South Africa.

Yanga Thungana*[1], Robert John Wilkinson[2,3,4], Zukiswa Zingela[5]

1. Department of Psychiatry and Behavioural Sciences, Walter Sisulu University, Mthatha, South Africa
2. Wellcome Center for Infectious Diseases Research in Africa, Institute of Infectious Disease and Molecular Med and Dept. Med. Univ. Cape Town, South Africa
3. Department of Infectious Disease, Imperial College, London, W12 0NN, UK
4. The Francis Crick Institute, London, NW1 1AT, UK
5. Executive Dean's Office, Faculty of Health Sciences, Nelson Mandela University, Port Elizabeth (now Gqeberha), South Africa

Correspondence address:

Yanga Thungana*

Department of psychiatry, Walter Sisulu University

Sisson street, Fort Gale, Mthatha, 5100

ythungana@wsu.ac.za

For peer review only

Abstract

Objectives: Tuberculosis (TB) remains prevalent despite the availability of effective anti-TB medications, and accumulating evidence suggests a high rate of mental disorders in people with TB. This is because TB and psychiatric disorders share several risk factors, such as poverty, homelessness, and substance use disorder. Moreover, psychiatric comorbidities in TB patients are associated with poor treatment outcomes.

This study explored the prevalence and clinical correlates of mental illness in outpatients with TB in a rural population in the Eastern Cape Province of South Africa.

Design*:* We conducted a cross-sectional survey over ten months.

Setting: Two primary care clinics at King Sabata Dalindyebo District (KSD), Mthatha, Eastern Cape, South Africa.

Participants: Patients receiving TB treatment in the two clinics.

Interventions: The Mini-International Neuropsychiatric Interview was used to screen for psychiatric disorders.

Primary and Secondary Outcome Measures: Rates of mental disorders in TB patients over a ten-month period. Variation in rates by sex, employment status, and HIV comorbidity.

Results: In a sample of 197 participants, most patients were male (62%), HIV-1 positive (65%), and screened positive for a mental disorder (82%) with anxiety (48%), depressive (38%), and substance use disorders (43%) being the most common psychiatric conditions. Female sex was associated with depressive disorders ($p = 0.005$), and males were more likely to have a substance use disorder ($p < 0.001$). Additionally, low education levels and unemployment were associated with depressive and anxiety disorders ($p < 0.05$).

Conclusions: Mental disorders are common in patients with tuberculosis, and mental health services need to be integrated in the management of patients with TB. Factors linked to mental disorders in this cohort, such as low education, gender and unemployment, may be

For peer review only

useful for compiling a risk profile to help identify those with TB who may require more intensive support for their mental health.

Strengths and limitations of this study

- All patients receiving TB treatment and attending the two clinics during the study period were included, allowing for findings that closely resemble reality.
- The MINI, a structured diagnostic interview evaluating several psychiatric disorders, allowed for a comprehensive assessment of mental disorders.
- The neuropsychiatric effects of COVID-19 could be a confounding factor to the high rates of mental illness in this study.
- The smaller study sample size could limit study power and generalizability of study findings.
- Using a psychiatric diagnostic scale without a follow-up clinical assessment could lead to an overestimation of the psychiatric disorder in this study.

For peer review only

Despite the availability of effective anti-tuberculosis drugs, tuberculosis (TB) remains one of the top ten causes of death worldwide, and it is among the leading causes of death from a single infectious agent.[1] South Africa is among the top eight countries that account for two-thirds of the global TB infections.[1] Additionally, in people infected with TB, there are high rates of mental disorders such as depression and anxiety disorders.[2–5] Depression is one of the most common comorbid psychiatric disorders in people with TB and is estimated to be 3-times higher than in patients without TB.[6–8] Conversely, people with psychiatric disorders may be at an increased risk of developing TB as well.[9–11]

There are several postulated reasons for the increased prevalence of mental illness in people with TB, including shared risk factors such as homelessness, HIV/AIDS, substance use, stigma, malnutrition, and poor socioeconomic status.[12–15] additionally, psychiatric comorbidity in TB is associated with poor treatment outcomes, disability, treatment failure, and poor quality of life due to poor health-seeking behaviours and challenges with adherence to medication in this cohort of patients.[16–18] Moreover, substance use, especially alcohol use, is prevalent in patients with tuberculosis and is thought to increase TB incidence, TB mortality, delayed presentation to health facilities, and poor adherence to TB treatment.[19–21] Furthermore, there is evidence that treating comorbid psychiatric disorders can improve TB outcomes.[22]

Although the syndemic relationship between TB and mental illness is described, there is often a lack of incorporation of mental health services in the management of patients with TB.[23] Additionally, in South Africa, limited studies have explored the prevalence of psychiatric disorders in people with TB. Therefore, the current study evaluated the prevalence and

For peer review only

clinical correlates of mental illness in patients with TB in two district clinics in a rural area in the Eastern Cape, South Africa.

METHODS

Sample and procedure

A cross-sectional study was conducted among TB patients attending two primary care clinics (Mthatha Gateway and Stanford Terrace) at King Sabata Dalindyebo District (KSD), Mthatha, Eastern Cape, South Africa. Both clinics offer general medical care, including TB screening and the management of patients with TB. All patients with TB who came for their TB treatment at these two clinics were identified, and those who gave a written consent were interviewed and 197 participants were included in the study. The interviews were conducted by a trained research assistant for ten months, from September 2020 to June 2021. The research assistant was trained over two weeks and continued to be supervised by a psychiatrist.

Measures

A data capture sheet was used to collect sociodemographic details, past psychiatric history, medical history including HIV status, and adherence to TB treatment. Data on whether a participant had defaulted their current TB treatment were also sought, although their responses were not verified through other means.

The Mini-International Neuropsychiatric Interview (MINI) was developed as a brief, structured diagnostic interview used as a measure to diagnose several psychiatric disorders

For peer review only

[24] It has demonstrated good psychometric properties as a tool to screen for several psychiatric disorders.[24–26] For instance, the specificity of the MINI is at least good to excellent (0.72 – 1.0) for the diverse conditions screened is used to evaluate.[25,27,28] Although the MINI contains over 100 questions and screens for 16 mental illnesses, and a personality disorder, it can be administered over a shorter period – approximately 20 – 30 minutes. The MINI is organised into diagnostic modules. For nearly all the modules, two to four screening questions are used to exclude a diagnosis if answered negatively. Conversely, positive responses to the screening questions lead to further inquiry about other diagnostic criteria. The MINI can be administered by clinicians or Lay interviewers after they undergo appropriate training. In this study, the research assistant administered the MINI to all study participants.

Patient and public involvement

No patient involvement in the design, recruitment or conduct of the study and results will not be disseminated to the participants directly.

Data analytical plan

Data were analysed using STATA/SE "(version 16.1 for Mac)". Descriptive statistics such as frequencies and proportions for categorical variables, median and interquartile range (IQR), or mean and standard deviation for continuous variables depending on the data skewness, were utilised to characterise the sample. Bivariate analyses were performed using either the Pearson's chi-square test or Wilcoxon rank-sum test with the significance level set at $P < 0.05$.

For peer review only

RESULTS

Most of the 197 participants were men (61.9%, n = 122) and the median age was 34 years (IQR 28-42) of age (**Table 1**). Most of the participants were single (70.6%, n = 139), had at least secondary education (56.9%, n= 112), and were unemployed (57.4%, n = 113). Unemployed participants were more likely to present with an anxiety disorder (p = 0.03), depressive disorder (p < 0.001), and obsessive-compulsive disorder (OCD) (p = 0.006). Although lower levels of education were associated with recurrent depressive disorder (P = 0.001) and OCD (p = 0.03), social anxiety disorder was more common in those with at least a secondary education (p = 0.02).

Table 1. Sociodemographic details of the participants (N=197)

	Number	Percentage
Sex		
Male	122	61.9%
Female	75	38.1%
Age		
Adolescents	4	2%
Adults	149	76%
Middle age	33	16.8%
Aged+	10	5.1%
Relationship status		
Single	139	70.6%
Married	44	22.3%
Divorced/separated	14	7.1%
Level of education		
Primary school	33	16.8%
High school	112	56.9%
Tertiary education	52	26.4%
Employment status		
Employed	84	42.6%

Unemployed	113	57.4%
Receiving state grant/pension	26	13.2%
**Monthly income[a]**		
Less than R2 500	127	64.5%
More than R2 500	70	35.5%
**HIV status**		
Negative	62	31.5%
Positive	128	64.97%
Unknown	7	3.6%
**Taking ART**		
Yes	99	77.3%
No	29	22.6%
**Disease classification**		
Pulmonary	153	77.7%
Extrapulmonary	44	22.3%
**Phase of TB treatment**		
Active	102	51.8%
Continuation	95	48.2%

Abbreviations: ART - Antiretroviral therapy; TB - Tuberculosis. [a] - SA Poverty line is R1 220

Medical history

The majority of the participants were HIV positive (65%, n = 128) (95% CI 58.0 - 71.4), most of whom were participants receiving antiretroviral therapy (77.3%, n = 99) (**Table 1**). Most HIV-positive (96.6%, n = 28) participants not on ART were still in the intensive phase of TB treatment. Moreover, most of the participants in the sample (77.7%, n = 153) had pulmonary TB, and more than half (51.8%, n = 102) were in the intensive phase of TB treatment. Additionally, a minority of patients (9.1%, n = 18) reported non-adherence to their TB treatment.

Mental disorders and TB in this sample

For peer review only

Most of the participants (82.7%, n = 163) screened positive for at least one psychiatric disorder, excluding substance use disorders (***Fig 1***). The most common psychiatric disorders were anxiety disorders (48.2%, n = 95), current major depressive episodes (40.6%, n = 80), unipolar depressive disorders (37.6%, n = 74), post-traumatic stress disorder (28.9%, n = 57), antisocial personality disorder (23.9%, n = 47), and bipolar spectrum disorders (19.3%, n = 38). Approximately a quarter (25.7%, n = 19) of the patients with unipolar depression currently had suicidal ideation. Additionally, more participants (47%) in the intensive phase of TB treatment had a current depressive episode compared to those in the continuation phase (34%); however, this was not statistically significant ($p = 0.056$). Noteworthily, female participants were more likely to have a depressive disorder than male participants ($p = 0.005$). In contrast, males were more likely to screen positive for an antisocial personality disorder ($p < 0.001$).

Substance use disorders

A considerable number of the participants had a substance use disorder (43.2%, n = 85), with more participants having an alcohol use disorder (40.1%, n = 79) than those having a substance use disorder other than alcohol (12.2%, n = 24). However, most of the 24 participants (79.2%, n = 19) with other substance use disorders also had alcohol use disorder. Nevertheless, both alcohol use disorders ($p = 0.001$) and other substance use disorders ($p = 0.001$) were more common among male participants.

DISCUSSION

In this study, we examined the prevalence of the psychiatric disorders in outpatients receiving TB treatment. We found a high prevalence of psychiatric disorders in patients with TB; depressive, anxiety, and substance use disorders were the most common mental disorders.

For peer review only - http://bmjopen.bmj.com/site/about/guidelines.xhtml

Additionally, there was a high HIV-1 comorbidity in our sample of patients with TB. Lastly, low levels of education and unemployment were associated with high psychiatric comorbidity.

The very high prevalence of mental disorders in our study could be due to several factors such as the assessment instrument used, a high HIV-1 comorbidity, the sociodemographic factors, or that it is a true reflection of the high psychiatric comorbidity in this population. For instance, the MINI covers a broader number of conditions than scales used in other studies and in patients with TB, mental disorders have been found to vary significantly depending on the psychiatric screening scale used.[29] Additionally, HIV-1 comorbidity in patients with TB is associated with higher mental morbidity.[30] Lastly, South Africa is a middle-income country with a very high TB burden; the personal socioeconomic consequences of TB infection might be amplified, thus leading to more pronounced mental disorders.[1,30] A case in point documented is that in TB patients, unemployment, poverty, and lower educational background are associated with higher psychiatric comorbidity, as was the case in our study.[6,31,32]

Our finding that anxiety and depressive disorders are the most common mental disorders in patients with TB is comparable to previous studies.[21,29,33,34] Moreover, as in our study, women with TB were more likely to suffer from depressive disorders than male patients.[29,30] Additionally, it has been reported that psychiatric disorders are more common in the earlier phase of TB infection and rates decline as patients recover from TB infection.[21] Our study showed a similar trend with more patients in the intensive phase of treatment having a current major depressive episode than those in the continuation phase; however, the difference did not reach statistical significance.

For peer review only - http://bmjopen.bmj.com/site/about/guidelines.xhtml

Alcohol use disorder was common in our study sample, which corroborates a finding also featured in previous literature highlighting high rates of alcohol use in patients with TB.[6,35–38] The importance of this finding is that alcohol use disorder might increase the risk of contracting TB.[20,21] Additionally, in those already infected with TB, alcohol abuse is associated with a lack of cooperation, poor adherence, higher levels of depression, and mortality.[19,20] Similar to previous studies, alcohol use disorders were more common among the male participants in our study.[37,39] However, the prevalence of alcohol use disorder (40%) in our study was higher than previously reported and could be because most participants were males, single, unemployed, and had lower education levels.[35,38,40]

Strengths and limitations of this study

The use of the MINI, a well-established structured diagnostic interview scale that covers a broader number of mental disorders adds to the strength of the study. Furthermore, all patients with TB attending the two clinics were admitted to the study with no exclusion criteria. The study was conducted during the COVID-19 pandemic, which possibly affected clinic attendance and TB screening; these factors could have contributed to the high prevalence of mental disorders.[41–44]. Along with this, and the small study sample could restrain the generalizability of the study results to other settings. Additionally, we did not conduct individual clinical assessments to confirm the diagnosis of the MINI, which could possibly increase the positive psychiatric diagnosis.

CONCLUSION

Our study showed a high prevalence of mental disorders among outpatients infected with TB. Thus, the study outcomes further highlight the need to integrate mental health services in

For peer review only

managing patients with TB at the primary care level, including screening of all new TB patients.

ACKNOWLEDGEMENTS

We would like to express our gratitude to our research assistant, Simthembile Lindani, who worked tirelessly and completed data collection despite the challenges of the COVID-19 pandemic. Additionally, acknowledge Dr Shwetha Suresh for her assistance with the research proposal for the grant application. We would also like to thank Editage (www.editage.com) for English language editing.

COMPETING INTERESTS

All authors have completed the ICMJE uniform disclosure form and declare no conflict of interests.

FUNDING

This research was Supported by the National Institutes of Health grant number (NIH U19AI115940). RJW is funded by Francis Crick Institute which receives funding from Wellcome (FC0010218), UKRI (FC0010218), and CRUK (FC00110218). For the purpose of open access the author has applied a CC-BY licence to any author accepted version of this manuscript.

DATA SHARING STATEMENT

The data (deidentified participants dataset) can be available upon reason request from the corresponding author.

AUTHORS' CONTRIBUTIONS

All authors were involved in the conception and design of the study. YT involved in gathering the data, data analysis , and all authors interpreted data analysis. YT wrote the manuscript, but all the authors revised and edited the manuscript. All authors provided their final approval for manuscript submission and agree to be acountable for all aspects of the

For peer review only

work. YT as the corresponding author confirms that all authors meet the authorship criteria and that no others meeting the criteria have been ommited.

ETHICS STATEMENT

Ethical approval for this study was obtained from the Walter Sisulu University Human Research Committee (protocol number 119/2019) and the South African Department of Health (EC_202006_009).

For peer review only

REFERENCES

[1] Global tuberculosis report. Geneva: World Health Organization; 2020. International Journal of Infectious Diseases 2021. https://doi.org/10.1016/j.ijid.2021.02.107.

[2] Mason PH, Sweetland AC, Fox GJ, Halovic S, Nguyen TA, Marks GB. Tuberculosis and mental health in the Asia-Pacific. Australasian Psychiatry 2016;24:553–5. https://doi.org/10.1177/1039856216649770.

[3] Koyanagi A, Vancampfort D, Carvalho AF, DeVylder JE, Haro JM, Pizzol D, et al. Depression comorbid with tuberculosis and its impact on health status: cross-sectional analysis of community-based data from 48 low- and middle-income countries. BMC Med 2017;15:209. https://doi.org/10.1186/s12916-017-0975-5.

[4] Doherty AM, Kelly J, McDonald C, O'Dywer AM, Keane J, Cooney J. A review of the interplay between tuberculosis and mental health. Gen Hosp Psychiatry 2013;35:398–406. https://doi.org/10.1016/j.genhosppsych.2013.03.018.

[5] Ruiz-Grosso P, Cachay R, de La Flor A, Schwalb A, Ugarte-Gil C. Association between tuberculosis and depression on negative outcomes of tuberculosis treatment: a systematic review and meta-analysis. PLoS ONE 2020;15:e0227472. https://doi.org/10.1371/journal.pone.0227472.

[6] Peltzer K, Naidoo P, Matseke G, Louw J, McHunu G, Tutshana B. Prevalence of post-traumatic stress symptoms and associated factors in tuberculosis (TB), TB retreatment and/or TB-HIV co-infected primary public health-care patients in three districts in South Africa. Psychol Health Med 2013;18:387–97. https://doi.org/10.1080/13548506.2012.726364.

[7] Peltzer K, Naidoo P, Matseke G, Louw J, Mchunu G, Tutshana B. Prevalence of psychological distress and associated factors in tuberculosis patients in public primary

For peer review only

care clinics in South Africa. BMC Psychiatry 2012;12:89. https://doi.org/10.1186/1471-244X-12-89.

[8] de Araújo GS, Pereira SM, dos Santos DN, Marinho JM, Rodrigues LC, Barreto ML. Common mental disorders associated with tuberculosis: a matched case-control study. PLoS ONE 2014;9:e99551. https://doi.org/10.1371/journal.pone.0099551.

[9] Mcquistlon L, Susser E. Tuberculosis infection among people with severe mental illness. Psychiatr Serv 1997;48:833–5. https://doi.org/10.1176/ps.48.6.833.

[10] Oh KH, Choi H, Kim EJ, Kim HJ, Cho SI. Depression and risk of tuberculosis: a nationwide populationbased cohort study. Inte J Tuberc Lung Dis 2017;21:804–9. https://doi.org/10.5588/ijtld.17.0038.

[11] Cheng KC, Liao KF, Lin CL, Lai SW. Increased risk of pulmonary tuberculosis in patients with depression: a cohort study in Taiwan. Frontiers in Psychiatry 2017;8:235. https://doi.org/10.3389/fpsyt.2017.00235.

[12] Pachi A, Bratis D, Moussas G, Tselebis A. Psychiatric morbidity and other factors affecting treatment adherence in pulmonary tuberculosis patients. Tuberculosis Research and Treatment 2013;2013:489865. https://doi.org/10.1155/2013/489865.

[13] Duarte EC, Bierrenbach AL, da Silva JB, Tauil PL, de Fátima Duarte E. Factors associated with deaths among pulmonary tuberculosis patients: a case control study with secondary data. Journal of Epidemiology and Community Health 2009;63:233–8. https://doi.org/10.1136/jech.2008.078972.

[14] Martens WHJ. A review of physical and mental health in homeless persons. Public Health Rev 2001;29:13–33.

[15] Oeltmann JE, Kammerer JS, Pevzner ES, Moonan PK. Tuberculosis and Substance Abuse in the United States, 1997-2006. Arch Intern Med 2009;169:189–97. https://doi.org/10.1001/archinternmed.2008.535.

For peer review only

[16] Simon GE, von Korff M, Lin E. Clinical and functional outcomes of depression treatment in patients with and without chronic medical illness. Psychol Med 2005;35:271–9. https://doi.org/10.1017/S0033291704003071.

[17] Ambaw F, Mayston R, Hanlon C, Medhin G, Alem A. Untreated depression and tuberculosis treatment outcomes, quality of life and disability, Ethiopia. Bull World Health Organ 2018;96:243–55. https://doi.org/10.2471/BLT.17.192658.

[18] Sweetland AC, Kritski A, Oquendo MA, Sublette ME, Norcini Pala A, Silva LRB, et al. Addressing the tuberculosis–depression syndemic to end the tuberculosis epidemic. Intern J Tuberc Lung Dis 2017;2:852–61. https://doi.org/10.5588/ijtld.16.0584.

[19] Lackey B, Seas C, van der Stuyft P, Otero L. Patient characteristics associated with tuberculosis treatment default: a cohort study in a high-incidence area of Lima, Peru. PLoS ONE 2015;10:e0128541. https://doi.org/10.1371/journal.pone.0128541.

[20] Imtiaz S, Shield KD, Roerecke M, Samokhvalov A v., Lönnroth K, Rehm J. Alcohol consumption as a risk factor for tuberculosis: meta-analyses and burden of disease. Eur Respir J 2017;50:1700216. https://doi.org/10.1183/13993003.00216-2017.

[21] Shyamala KK, Sharadha Naveen R, Khatri B. Depression: a neglected comorbidity in patients with tuberculosis. J Assoc Physicians India 2018;66:18–21.

[22] Acha J, Sweetland A, Guerra D, Chalco K, Castillo H, Palacios E. Psychosocial support groups for patients with multidrug-resistant tuberculosis: five years of experience. Global Public Health 2007;2:404–17. https://doi.org/10.1080/17441690701191610.

[23] Walker IF, Baral SC, Wei X, Huque R, Khan A, Walley J, et al. Multidrug-resistant tuberculosis treatment programmes insufficiently consider comorbid mental disorders. Intern J Tuberc Lung Dis 2017;21:603–9. https://doi.org/10.5588/ijtld.17.0135.

For peer review only

[24] Sheehan D v., Lecrubier Y, Sheehan KH, Amorim P, Janavs J, Weiller E, et al. The Mini-International Neuropsychiatric Interview (M.I.N.I.): The development and validation of a structured diagnostic psychiatric interview for DSM-IV and ICD-10. J Clin Psychiatry, vol. 59, 1998, p. 22–33.

[25] Lecrubier Y, Sheehan D v., Weiller E, Amorim P, Bonora I, Sheehan KH, et al. The Mini International Neuropsychiatric Interview (MINI): a short diagnostic structured interview: Reliability and validity according to the CIDI. Eur Psychiatry 1997;12:224–31. https://doi.org/10.1016/S0924-9338(97)83296-8.

[26] Sheehan D v., Lecrubier Y, Sheehan KH, Janavs J, Weiller E, Keskiner A, et al. The validity of the Mini International Neuropsychiatric Interview (MINI) according to the SCID-P and its reliability. Eur Psychiatry 1997;12:232–41. https://doi.org/10.1016/S0924-9338(97)83297-X.

[27] Otsubo T, Tanaka K, Koda R, Shinoda J, Sano N, Tanaka S, et al. Reliability and validity of Japanese version of the Mini-International Neuropsychiatric Interview. Psychiatry Clin Neurosci 2005;59:517–26. https://doi.org/10.1111/j.1440-1819.2005.01408.x.

[28] Sheehan D v., Sheehan KH, Shytle RD, Janavs J, Bannon Y, Rogers JE, et al. Reliability and validity of the mini international neuropsychiatric interview for children and adolescents (MINI-KID). J Clin Psychiatry 2010;71:313–26. https://doi.org/10.4088/JCP.09m05305whi.

[29] Duko B, Bedaso A, Ayano G. The prevalence of depression among patients with tuberculosis: a systematic review and meta-analysis. Ann Gen Psychiatry 2020;19:30. https://doi.org/10.1186/s12991-020-00281-8.

For peer review only

[30] Theron G, Peter J, Zijenah L, Chanda D, Mangu C, Clowes P, et al. Psychological distress and its relationship with non-adherence to TB treatment: A multicentre study. BMC Infect Dis 2015;15:253. https://doi.org/10.1186/s12879-015-0964-2.

[31] Xu M, Markström U, Lyu J, Xu L. Survey on tuberculosis patients in rural areas in China: tracing the role of stigma in psychological distress. Int J Environ Res Public Health 2017;14:1171. https://doi.org/10.3390/ijerph14101171.

[32] Chen X, Wu R, Xu J, Wang J, Gao M, Chen Y, et al. Prevalence and associated factors of psychological distress in tuberculosis patients in Northeast China: a cross-sectional study. BMC Infect Dis 2021;21:563. https://doi.org/10.1186/s12879-021-06284-4.

[33] Husain MO, Dearman SP, Chaudhry IB, Rizvi N, Waheed W. The relationship between anxiety , depression and illness perception in tberculosis patients in Pakistan. Clinical Practice and Epidemiology in Mental Health 2008;4:4.

[34] Janse Van Rensburg A, Dube A, Curran R, Ambaw F, Murdoch J, Bachmann M, et al. Comorbidities between tuberculosis and common mental disorders: a scoping review of epidemiological patterns and person-centred care interventions from low-to-middle income and BRICS countries. Infect Dis Poverty 2020;9:4. https://doi.org/10.1186/s40249-019-0619-4.

[35] Suhadev M, Thomas BE, Raja Sakthivel M, Murugesan P, Chandrasekaran V, Charles N, et al. Alcohol use disorders (AUD) among tuberculosis patients: a study from Chennai, South India. PLoS ONE 2011;6:e19485. https://doi.org/10.1371/journal.pone.0019485.

[36] Thapa P, Kamath R, Shetty BK, Monteiro A, Sekaran VC. Prevalence and associated Factors of alcoholism among tuberculosis patients in Udupi Taluk, Karnataka, India: a cross sectional study. J Nepal Health Res Counc 2014;12:177–81.

For peer review only

[37] Peltzer K, Louw J, Mchunu G, Naidoo P, Matseke G, Tutshana B. Hazardous and harmful alcohol use and associated factors in tuberculosis public primary care patients in South Africa. IntJ Environ Res Public Health 2012;9:3245–57. https://doi.org/10.3390/ijerph9093245.

[38] Necho M, Tsehay M, Seid M, Zenebe Y, Belete A, Gelaye H, et al. Prevalence and associated factors for alcohol use disorder among tuberculosis patients: a systematic review and meta-analysis study. Subst Abuse Treat Prev Policy 2021;16:2. https://doi.org/10.1186/s13011-020-00335-w.

[39] Veerakumar AM, Sahu SK, Sarkar S, Kattimani S, Govindarajan S. Alcohol use disorders among pulmonary tuberculosis patients under RNTCP in urban Pondicherry, India. Indian J Tuberc 2015;62:171–7. https://doi.org/10.1016/j.ijtb.2015.07.017.

[40] O'Connell R, Chishinga N, Kinyanda E, Patel V, Ayles H, Weiss HA, et al. Prevalence and correlates of alcohol dependence disorder among TB and HIV infected patients in Zambia. PLoS ONE 2013;8:e74406. https://doi.org/10.1371/journal.pone.0074406.

[41] Taquet M, Geddes JR, Husain M, Luciano S, Harrison PJ. 6-month neurological and psychiatric outcomes in 236 379 survivors of COVID-19: a retrospective cohort study using electronic health records. Lancet Psychiatry 2021;8:416–27. https://doi.org/10.1016/S2215-0366(21)00084-5.

[42] Lebina L, Dube M, Hlongwane K, Brahmbatt H, Lala SG, Reubenson G, et al. Trends in paediatric tuberculosis diagnoses in two South African hospitals early in the COVID-19 pandemic. S Afr Med J 2020;110:1149–50. https://doi.org/10.7196/SAMJ.2020.v110i12.15386.

[43] Kwak N, Hwang SS, Yima AJ. Effect of COVID-19 on tuberculosis notification, South Korea. Emerging Infectious Diseases 2020;26:2506–8. https://doi.org/10.3201/EID2610.202782.

For peer review only

[44] Hogan AB, Jewell BL, Sherrard-Smith E, Vesga JF, Watson OJ, Whittaker C, et al. Potential impact of the COVID-19 pandemic on HIV, tuberculosis, and malaria in low-income and middle-income countries: a modelling study. Lancet Glob Health 2020;8:e1132-41. https://doi.org/10.1016/S2214-109X(20)30288-6.

Figure 1: Common mental disorder in TB patients (N =197)

For peer review only

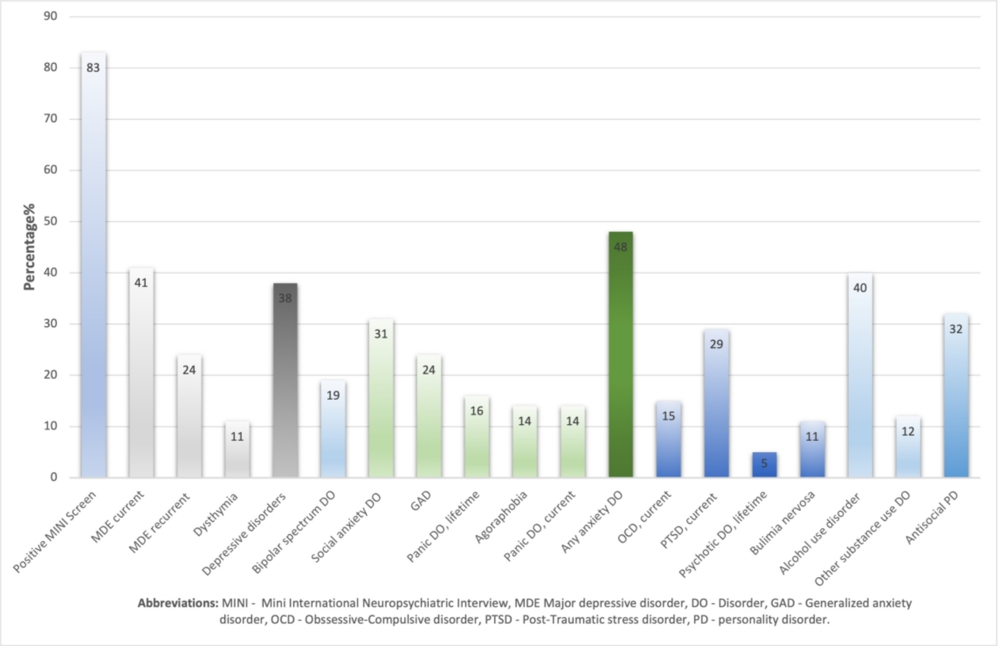

Figure 1: Common mental disorders in TB patients (N =197).

239x155mm (300 x 300 DPI)

BMJ Open

The Co-Morbidity of Mental Ill-Health in Tuberculosis Patients under Treatment in a Rural Province of South Africa – A cross-sectional survey.

Journal:	*BMJ Open*
Manuscript ID	bmjopen-2021-058013.R1
Article Type:	Original research
Date Submitted by the Author:	30-Jun-2022
Complete List of Authors:	Thungana, Yanga; Walter Sisulu University, Department of Psychiatry and Behavioural Sciences<br>Wilkinson, Robert; Francis Crick Institute, Pathology; Imperial College London<br>Zingela, Zukiswa; Nelson Mandela University, Executive Dean's Office, Faculty of Health Sciences
**Primary Subject Heading**:	Mental health
Secondary Subject Heading:	Infectious diseases, Mental health, Public health
Keywords:	Tuberculosis < INFECTIOUS DISEASES, Substance misuse < PSYCHIATRY, Adult psychiatry < PSYCHIATRY, PUBLIC HEALTH

SCHOLARONE™
Manuscripts

I, the Submitting Author has the right to grant and does grant on behalf of all authors of the Work (as defined in the below author licence), an exclusive licence and/or a non-exclusive licence for contributions from authors who are: i) UK Crown employees; ii) where BMJ has agreed a CC-BY licence shall apply, and/or iii) in accordance with the terms applicable for US Federal Government officers or employees acting as part of their official duties; on a worldwide, perpetual, irrevocable, royalty-free basis to BMJ Publishing Group Ltd ("BMJ") its licensees and where the relevant Journal is co-owned by BMJ to the co-owners of the Journal, to publish the Work in this journal and any other BMJ products and to exploit all rights, as set out in our licence.

The Submitting Author accepts and understands that any supply made under these terms is made by BMJ to the Submitting Author unless you are acting as an employee on behalf of your employer or a postgraduate student of an affiliated institution which is paying any applicable article publishing charge ("APC") for Open Access articles. Where the Submitting Author wishes to make the Work available on an Open Access basis (and intends to pay the relevant APC), the terms of reuse of such Open Access shall be governed by a Creative Commons licence – details of these licences and which Creative Commons licence will apply to this Work are set out in our licence referred to above.

Other than as permitted in any relevant BMJ Author's Self Archiving Policies, I confirm this Work has not been accepted for publication elsewhere, is not being considered for publication elsewhere and does not duplicate material already published. I confirm all authors consent to publication of this Work and authorise the granting of this licence.

For peer review only

The Co-Morbidity of Mental Ill-Health in Tuberculosis Patients under Treatment in a Rural Province of South Africa – A cross-sectional survey.

Yanga Thungana*[1], Zukiswa Zingela[1], Robert John Wilkinson[2,3,4]

1. Department of Psychiatry and Behavioural Sciences, Walter Sisulu University, Mthatha, South Africa
2. Wellcome Center for Infectious Diseases Research in Africa, Institute of Infect. Disease and Mol. Med and Dept. med. Univ. Cape Town, South Africa
3. Department of Infectious Disease, Imperial College, London, W12 ONN, UK
4. The Francis Crick Institute, London, NW1 IAT, UK

Correspondence address:

Yanga Thungana*

Department of psychiatry, Walter Sisulu University

Sisson street, Fort Gale, Mthatha, 5100

ythungana@wsu.ac.za

For peer review only

Abstract

Objectives: Tuberculosis (TB) remains prevalent despite the availability of effective anti-TB medications, and accumulating evidence suggests a high rate of mental disorders in people with TB. This is because TB and psychiatric disorders share several risk factors, such as poverty, homelessness, and substance use disorder. Moreover, psychiatric comorbidities in TB patients are associated with poor treatment outcomes. This study explored the psychiatric comorbidity and its clinical in individuals receiving TB treatment.

Design*:* A cross-sectional survey over ten months.

Setting: Two primary care clinics at King Sabata Dalindyebo District (KSD), Mthatha, Eastern Cape, South Africa.

Participants: Patients receiving TB treatment in the two clinics.

Interventions: The Mini-International Neuropsychiatric Interview was used to screen for psychiatric disorders.

Primary and Secondary Outcome Measures: Rates of mental disorders in TB patients over a ten-month period. Variation in rates by sex, employment status, and HIV comorbidity.

Results: In a sample of 197 participants, most patients were male (62%) and screened positive for a mental disorder (82%) with anxiety (48%), depression (38%), and substance use disorders (43%) being the most common psychiatric conditions. On average, individuals had 4 (SD 2) mental disorders. Females had higher rates of depression ($p = 0.005$) and nonadherence to TB treatment ($p = 0.003$), and alcohol use disorder was more common in males ($p< 0.001$) and in those nonadherent to TB treatment. Additionally, low education levels and unemployment were associated with depressive and anxiety disorders ($p < 0.05$).

Conclusions: Mental disorders are common in patients with tuberculosis, and mental health services need to be integrated into the management of patients with TB. Factors linked to mental disorders in this cohort, such as low education, gender, and unemployment, may be

For peer review only

useful for compiling a risk profile to help identify those with TB who may require more intensive support for their mental health.

For peer review only

Despite the availability of effective anti-tuberculosis drugs, tuberculosis (TB) remains one of the top ten causes of death worldwide, and it is among the leading causes of death from a single infectious agent. [1] South Africa is among the top eight countries that account for two-thirds of the global TB infections. [1] Additionally, in people infected with TB, there are high rates of mental disorders such as depression and anxiety disorders. [2–5] Depression is one of the most common comorbid psychiatric disorders in people with TB and is estimated to be 3-times higher than in patients without TB. [6–8] Conversely, people with psychiatric disorders may be at an increased risk of developing TB as well. [9–11]

There are several postulated reasons for the increased prevalence of mental illness in people with TB, including shared risk factors such as homelessness, HIV/AIDS, substance use, stigma, malnutrition, and poor socioeconomic status. [12–15] Psychiatric comorbidity in TB is associated with poor treatment outcomes, disability, treatment failure, and poor quality of life due to poor health-seeking behaviours and challenges with adherence to medication in this cohort of patients. [16–18] Substance use, especially alcohol use, is prevalent in patients with tuberculosis and is thought to increase TB incidence, TB mortality, delayed presentation to health facilities, and poor adherence to TB treatment. [19–21] Furthermore, there is evidence that treating comorbid psychiatric disorders can improve TB outcomes. [22]

Although the relationship between TB and mental illness is described, there is often a lack of incorporation of mental health services in the management of patients with TB. [23] Additionally, in South Africa, limited studies have explored in detail the prevalence of psychiatric disorders in people with TB. The value of this study is in the use of a different psychiatry questionnaire (Mini-International Neuropsychiatric Interview) that is more extensive and evaluates many more different psychiatric disorders compared to previous

For peer review only

studies evaluating mental disorders in TB patients. Also, unique to our study is that the MINI is observer administered compared to the commonly used self-report questionnaires. Therefore, we sought to investigate the point prevalence of comorbid mental illness and its clinical correlates in patients receiving TB treatment in two district clinics in a rural area in the Eastern Cape, South Africa.

METHODS

Sample and procedure

A cross-sectional study was conducted among TB patients attending two primary care clinics (Mthatha Gateway and Stanford Terrace) at King Sabata Dalindyebo District (KSD), Mthatha, Eastern Cape, South Africa. Both clinics offer general medical care, including TB screening and the management of patients with TB. The study was conducted over a ten month period, from September 2020 to June 2021. Participant included in the study were all patients currently diagnosed and receiving TB treatment who attended the two clinics during the study period. In these clinics, each morning a nurse holds a short meeting with patients attending the clinic on the day to triage and guide them on accessing services. It is in these morning meetings that the nurse would identify all patients coming to collect tuberculosis treatment and would inform the research assistant who then approached each patient individually. Due to the coronavirus (Covid-19) pandemic and applicable South African lockdown restrictions at the time, the total number of patients attending clinics was significantly reduced.

For peer review only

The RA would explain the research study to each patient individually and obtain written informed consent. Excluded from the research were participants under the age of 18 years and those who could not give informed consent. A total of 197 participants were approached by the RA, all provided written consent and thus were included in the study.

Data collection

The research assistant who had prior experience in mental health research and was trained on the use of the MINI over ten working days by a psychiatrist. The training was held for 90 minutes each day regarding how to use the different modules of the MINI and an explanation of the various psychiatric terms used in the MINI. The last two sessions were role-playing interviews to assess the RA's level of understanding of the MINI. Lastly, the RA received supervision throughout the period of data collection from the lead author.

The English version of the MINI was administered in English to all study participants. The research assistant was instructed to read the questions slowly, repeat them as necessary, and encourage the patients to seek clarity on any question that is not entirely clear to them. The RA was instructed to schedule at least an hour to accommodate those slow in answering the numerous questions of the MINI. Lastly, when a patient did not understand a question, the RA was allowed to explain it in the patient's primary language.

Measures

A data capture sheet was used to collect sociodemographic details, past psychiatric history, medical history including HIV status, and adherence to TB treatment. Data on whether a

For peer review only

participant had defaulted their current TB treatment were also sought from the patient, although their responses were not verified through other means. The patients were asked whether they had stopped taking the TB treatment for a period of at least 7 consecutive days.

The Mini-International Neuropsychiatric Interview (MINI) was developed as a brief, structured diagnostic interview used as a measure to diagnose several psychiatric disorders. [24] It has demonstrated good psychometric properties as a tool to screen for several psychiatric disorders. [24–26] For instance, the specificity of the MINI is at least good to excellent (0.72 – 1.0) for the diverse conditions screened is used to evaluate. [25,27,28] Although the MINI contains over 100 questions and screens for 16 mental illnesses, and a personality disorder, it can be administered over a short period – approximately 20 – 30 minutes. The MINI is organised into diagnostic modules. For nearly all the modules, two to four screening questions are used to exclude a diagnosis if answered negatively. Conversely, positive responses to the screening questions lead to further inquiry about other diagnostic criteria. The MINI modules included in our study were major depressive disorder (current and recurrent), dysthymia, manic & hypomanic episodes (current and past), panic disorder (current and lifetime), agoraphobia, social phobia, obsessive-compulsive disorder, post-traumatic stress disorder, alcohol abuse & dependence, non-alcohol substance use & dependence, psychotic disorders (current and lifetime), anorexia nervosa, bulimia nervosa, generalized anxiety disorder, and antisocial personality disorder.

Patient and public involvement

There was no involvement of patients in the design, recruitment, and conduct of the study. The study results will not be distributed to the individual participants, but the published paper will be available in the participating clinics.

For peer review only - http://bmjopen.bmj.com/site/about/guidelines.xhtml

Data analytical plan

Data were analysed using STATA/SE "(version 16.1 for Mac)". Descriptive statistics such as frequencies and proportions for categorical variables, median and interquartile range (IQR), or mean and standard deviation for continuous variables depending on the data skewness were utilised to characterise the sample. Inferential statistics were applied to evaluate the association between different variables. Firstly, to assess associations between categorical variables, we performed either the Pearson's chi-square test or Fischer's exact test, depending on the number of scored items under each variable. Secondly, for the continuous variables, we first assessed for normality using the Skewness and Kurtosis test (*sktest*) with a $p > 0.05$ indicating normal distribution. Therefore, due to the skewness of our data, we used either the Wilcoxon rank-sum test or the Kruskal-Wallis test to evaluate associations between numerical and categorical variables. The significance level was set at $P < 0.05$ for all the bi-variate hypothesis testing.

Ethics approval

Ethical approval for this study was obtained from the Walter Sisulu University Human Research Committee (protocol number 119/2019) and the South African Department of Health (EC_202006_009).

RESULTS

For peer review only

Most of the 197 participants were men (61.9%, n = 122), and the median age was 34 years (IQR 28-42) of age (**Table 1**). Most of the participants were single (70.6%, n = 139), had at least secondary education (56.9%, n= 112), and were unemployed (57.4%, n = 113). We did not find any statistically significant associations between age and the different psychiatric disorders. For instance, the adult group (44%, n = 66) had the highest proportion of participants with an alcohol use disorder compared to other age groups, but it was not statistically significant ($p = 0.055$). Unemployed participants were more likely to have an anxiety disorder ($p = 0.03$), depressive disorder ($p < 0.001$), and obsessive-compulsive disorder (OCD) ($p = 0.006$). Moreover, individuals earning less than R2500.00 a month had a higher proportion of depressive disorders (74.7%, $p < 0.001$), social phobia (75.4%, $p = 0.03$), and agoraphobia (85.7%, $p = 0.01$) than those earning more than R2500.00. Lower levels of education were associated with recurrent depressive disorder ($P = 0.001$), lifetime panic disorder ($p = 0.02$), ongoing generalized anxiety disorder ($p = 0.02$), and current OCD ($p = 0.03$). But social anxiety disorder was more common in those with at least a high school education ($p = 0.02$).

Table 1. Sociodemographic details of the participants (N=197)

	Number	Percentage
Sex		
Male	122	61.9%
Female	75	38.1%
Age		
Adolescents	4	2%
Adults	149	76%
Middle age	33	16.8%
Aged+	10	5.1%
Relationship status		
Single	139	70.6%

For peer review only

Married	44	22.3%
Divorced/separated	14	7.1%
**Level of education**		
Primary school	33	16.8%
High school	112	56.9%
Tertiary education	52	26.4%
**Employment status**		
Employed	84	42.6%
Unemployed	113	57.4%
Receiving state grant/pension	26	13.2%
**Monthly income[a]**		
Less than R2 500	127	64.5%
More than R2 500	70	35.5%
**HIV status**		
Negative	62	31.5%
Positive	128	64.97%
Unknown	7	3.6%
**Taking ART**		
Yes	99	77.3%
No	29	22.6%
**Disease classification**		
Pulmonary	153	77.7%
Extrapulmonary	44	22.3%
**Phase of TB treatment**		
Active	102	51.8%
Continuation	95	48.2%

Abbreviations: ART - Antiretroviral therapy; TB - Tuberculosis. [a] - SA Poverty line is R1 220

Medical history

The majority of the participants were HIV positive (65%, n = 128) (95% CI 58.0 - 71.4) with significantly higher proportion of males (78.4%, n = 58) having HIV than females (60.7, n = 71) participants (p = 0.01). Most participants with a HIV diagnosis were receiving

For peer review only

antiretroviral therapy (77.3%, n = 99) (**Table 1**). Most HIV-positive (96.6%, n = 28) participants not on ART were still in the intensive phase of TB treatment. Moreover, most of the participants in the sample (77.7%, n = 153) had pulmonary TB, and more than half (51.8%, n = 102) were in the intensive phase of TB treatment. Only a minority of patients (9.1%, n = 18) reported non-adherence to their TB treatment. The majority of participants reporting non-adherence to TB treatment were HIV positive (77.8%, p = 0.4), still in the intensive phase (55.6%, p = 0.8) of TB treatment, unemployed (55.6%, p = 0.8), and assessed to have a mental disorder (88.9%, p = 0.4) but these did not reach statistical significance. However, being female (p = 0.003) and having an alcohol use disorder (p = 0.001) was significantly associated with non-adherence to TB treatment.

Mental disorders

Most of the participants (82.7%, n = 163) screened positive for at least one psychiatric disorder, excluding substance use disorders (***Fig 1***), and the proportion of males (82.1%, n = 101) with a positive screen was similar to that of female (84%, n = 63) participants. Furthermore, the HIV status of the participants was not associated with screening outcomes for psychiatric disorders with those HIV negative almost screening equally positive for a psychiatric disorder as those who were HIV positive (80.7% vs. 85.2%, p = 0.4). There was a comparable proportion of individuals assessed to have a psychiatric diagnosis in those in the intensive phase (82.5%, n = 85) and a continuation phase (83.2%, n = 79) of TB treatment.

The most common psychiatric disorders were anxiety disorders (48.2%, n = 95), current major depressive episodes (40.6%, n = 80), unipolar depression (37.6%, n = 74), post-traumatic stress disorder (28.9%, n = 57), antisocial personality (23.9%, n = 47), and bipolar

For peer review only

spectrum conditions (19.3%, n = 38). Approximately a quarter (25.7%, n = 19) of the patients with unipolar depression currently had suicidal ideation. The patients with a positive diagnosis of a mental disorder had an average of 3.5 (SD 2.3) disorders excluding substance use disorders. Also, patients tended to have more than one anxiety disorder with an average of 2 (SD 1) disorders per individual.

More participants (47%) in the intensive phase of TB treatment had a current depressive episode compared to those in the continuation phase (34%); however, this was not statistically significant ($p = 0.056$). Noteworthily, female participants were more likely to have a depressive disorder than male participants ($p = 0.005$). In contrast, males were more likely to screen positive for an antisocial personality disorder ($p < 0.001$).

Substance use disorders

A considerable number of the participants had a substance use disorder (43.2%, n = 85), with more participants having an alcohol use disorder (40.1%, n = 79) than those having a substance use disorder other than alcohol (12.2%, n = 24). However, most of the 24 participants (79.2%, n = 19) with other substance use disorders also had alcohol use disorder. Nevertheless, both alcohol use disorders ($p = 0.001$) and other substance use disorders ($p = 0.001$) were more common among male participants. In this sample, there was no significant association between HIV status and substance use disorders.

DISCUSSION

For peer review only

In this study, we examined the psychiatric comorbidity in outpatients receiving TB treatment. We found a higher rate of mental illness in patients with TB; depression, anxiety, and substance use disorders were the most common mental disorders. Additionally, those with mental illness approximately had 4 (SD 2) lifetime psychiatric disorders excluding substance use. Alcohol use disorder was prevalent in our study participants and had a significant association with the male sex and poor adherence to TB treatment. Female sex was associated with depressive disorders and nonadherence to treatment. Lastly, low levels of education and unemployment were associated with a higher rate of depressive, anxiety, and obsessive-compulsive disorders.

The prevalence of psychiatric disorders in patients with tuberculosis varies greatly among different studies from less than 20% to about 80% with a trend of higher rates in lower-middle-income countries (LMIC) [21,29–31]. In South Africa (SA), the prevalence of mental comorbidity in TB patients is similar to other LMIC. For instance, the prevalence of depression is documented to be 64.3%, and PTSD at 29.6%.[6,32] But higher rates of psychological distress (81%) have been reported in SA.[6,7] In our study, we found an overall higher rate of mental disorders (approx. 83%), but the rates of PTSD and depression were similar and lower to previous studies, respectively.[6,32] The major variation in prevalence in our study could be due to several factors such as the assessment instrument used, a high HIV comorbidity, the sociodemographic factors, or that it is a true reflection of the high psychiatric comorbidity in this population. For instance, HIV comorbidity in patients with TB is associated with higher mental morbidity.[33] Also, South Africa is a middle-income country with a very high TB burden; the personal socioeconomic consequences of TB infection might be amplified, thus leading to more pronounced mental disorders. [1,33] A case in point documented is that in TB patients, unemployment, poverty, and lower

For peer review only

educational background are associated with higher psychiatric comorbidity, as was the case in our study. [6,34,35]

Furthermore, the MINI covers a broader number of conditions than scales used in other studies, and in patients with TB, mental disorders have been found to vary significantly depending on the psychiatric screening scale used. [36] Compared to previous studies that tended to assess broader categories of mental disturbance such as anxiety or psychological distress, we were able to screen for several individual psychiatric diagnoses. Thus, in addition to describing the presence of mental illness, we were able to assess the number of disorders experienced by each individual and we found a tendency for participants to have more than one psychiatric disorder with an average of approx. 4 (SD 2) disorders per individual excluding substance use disorders.

In this study, we did not find a statistically significant relationship between HIV status and having a mental disorder. However, the bidirectional relationship between HIV and Mental disorders is well demonstrated.[37,38] Therefore, the finding of increased psychiatric disorders in PLWH is not surprising. [39–44] However, the prevalence of mental illness in PLWH varies significantly from as low as below 10% to as high as over 80%. [29,43,45–47] The wide range in the prevalence could be due to the different study designs, study settings, and measurement instruments used in these studies. Moreover, not all studies have found an increased rate of mental illness in PLWH. [48] The current study also did not demonstrate a relationship between HIV and adherence to TB treatment in our study. But previous studies in PLWH have demonstrated increased non-compliance to treatment with psychiatric comorbidity.[49–51] However, a recent sub-Saharan systemic review could not conclude

For peer review only

whether psychiatric comorbidity is associated with treatment non-adherence in HIV-positive patients.[52] Nevertheless, our study only focused on adherence to TB treatment.

We found a slightly higher adherence rate to TB treatment (91%) in the current study. Previous studies have documented adherence rates between 70% and 89%. [53–57] The higher rates of treatment adherence in our study could be due to our methodology as we solely relied on the patient's report and did investigate it further. Non-adherence to treatment in patients with TB can be due to several factors including HIV seropositivity, substance use, distance to health facilities, male gender, running out of medication, medication factors, being in the continuation phase of chemotherapy, and stigma. [53,55,56,58] Additionally, psychiatric comorbidity has been described as another factor with a negative impact on compliance with TB treatment.[5,33,59,60] Also, substance use is associated with poor adherence to TB treatment,[60–63] as was the case in our study. Contrary to findings in the current study, previous studies have found better adherence to tuberculosis treatment among female participants.[56,58] Furthermore, the proportion of participants with psychiatric disorders was comparable between those in the intensive phase and continuation phase of chemotherapy. But the continuation phase of TB treatment is previously associated with a higher rate of mental illness.[57,64,65]

Our finding that anxiety and depressive disorders are the most common mental disorders in patients with TB is comparable to previous studies. [21,36,66,67] Moreover, as in our study, women with TB were more likely to suffer from depressive disorders than male patients.[33,36] It has been reported that psychiatric disorders are more common in the earlier phase of TB infection and rates decline as patients recover from TB infection.[21] Our study showed a similar trend with more patients in the intensive phase of treatment having a current

For peer review only

major depressive episode than those in the continuation phase; however, the difference did not reach statistical significance.

Alcohol use disorder was common in our study sample, which corroborates a finding also featured in previous literature highlighting high rates of alcohol use in patients with TB.[6,68–71] Alcohol use disorder is documented to increase the risk of contracting TB, so the high rates of substance use could also be the consequence of premorbid alcohol in the participants.[20,21] In those already infected with TB, alcohol abuse is associated with a lack of cooperation, poor adherence, higher levels of depression, and mortality.[19,20] Similar to previous studies, alcohol use disorders were more common among the male participants in our study.[70,72] However, the prevalence of alcohol use disorder (40%) in our study was higher than previously reported and could be because most participants were males, single, unemployed, and had lower education levels.[68,71,73]

Strengths and limitations of this study

The use of the MINI, a well-established structured diagnostic interview scale that covers a broader number of mental disorders adds to the strength of the study. Furthermore, all patients with TB attending the two clinics were admitted to the study with no exclusion criteria.

The study was conducted during the COVID-19 pandemic, which possibly affected clinic attendance and TB screening; these factors could have contributed to the high prevalence of mental disorders.[74–77] Along with this, and the small study sample could restrain the generalizability of the study results to other settings. Additionally, we did not conduct individual clinical assessments to confirm the diagnosis of the MINI, which could possibly increase the positive psychiatric diagnosis. Lastly, the MINI was administered in English to

For peer review only

participants to whom English is not their primary language and this might have affected the accuracy of the patient’s responses.

CONCLUSION

Our study showed an increased frequency and number of mental disorders among individuals receiving TB treatment. Moreover, as in previous studies, we found substance use disorders to be prevalent in TB patients. Thus, the study outcomes further highlight the need to integrate mental health services in managing patients with TB at the primary care level, including screening of all new TB patients.

ACKNOWLEDGEMENTS

We would like to express our gratitude to our research assistant, Simthembile Lindani, who worked tirelessly and completed data collection despite the challenges of the COVID-19 pandemic. We would like to thank Editage (www.editage.com) for English language editing.

COMPETING INTERESTS

All authors have completed the ICMJE uniform disclosure form and declare no conflict of interest.

FUNDING

This research was Supported by the National Institutes of Health grant number (NIH U19AI115940). RJW is supported by the Francis Crick Institue which receives funding from Wellcome (FC0010218), Cancer Research UK (FC0010218), and the Medical Research Council (FC0010218). For the purpose of open access, the authors have applied a CC-BY public copyright to any author-accepted manuscript arising from this submission.

DATA SHARING STATEMENT

For peer review only

The data (deidentified participants dataset) can be available upon reasonable request from the corresponding author.

AUTHORS' CONTRIBUTIONS

All authors were involved in the conception and design of the study. YT involved in gathering the data, data analysis , and all authors interpreted data analysis. YT wrote the manuscript, but all the authors revised the manuscript. All authors provided their final approval for manuscript submission and agree to be acountable for all aspet of the work. YT as the corresponding author confirms that all authors meet the authorship criteria and that no others meeting the criteria have been omitted.

For peer review only

REFERENCES

[1] Global tuberculosis report. Geneva: World Health Organization; 2020. International Journal of Infectious Diseases 2021. https://doi.org/10.1016/j.ijid.2021.02.107.

[2] Mason PH, Sweetland AC, Fox GJ, Halovic S, Nguyen TA, Marks GB. Tuberculosis and mental health in the Asia-Pacific. Australasian Psychiatry 2016;24. https://doi.org/10.1177/1039856216649770.

[3] Koyanagi A, Vancampfort D, Carvalho AF, DeVylder JE, Haro JM, Pizzol D, et al. Depression comorbid with tuberculosis and its impact on health status: Cross-sectional analysis of community-based data from 48 low- and middle-income countries. BMC Medicine 2017;15. https://doi.org/10.1186/s12916-017-0975-5.

[4] Doherty AM, Kelly J, McDonald C, O'Dywer AM, Keane J, Cooney J. A review of the interplay between tuberculosis and mental health. General Hospital Psychiatry 2013;35:398–406. https://doi.org/10.1016/j.genhosppsych.2013.03.018.

[5] Ruiz-Grosso P, Cachay R, de La Flor A, Schwalb A, Ugarte-Gil C. Association between tuberculosis and depression on negative outcomes of tuberculosis treatment: a systematic review and meta-analysis. PLoS ONE 2020;15:e0227472. https://doi.org/10.1371/journal.pone.0227472.

[6] Peltzer K, Naidoo P, Matseke G, Louw J, McHunu G, Tutshana B. Prevalence of post-traumatic stress symptoms and associated factors in tuberculosis (TB), TB retreatment and/or TB-HIV co-infected primary public health-care patients in three districts in South Africa. Psychol Health Med 2013;18:387–97. https://doi.org/10.1080/13548506.2012.726364.

[7] Peltzer K, Naidoo P, Matseke G, Louw J, Mchunu G, Tutshana B. Prevalence of psychological distress and associated factors in tuberculosis patients in public primary

For peer review only

care clinics in South Africa. BMC Psychiatry 2012;12:89. https://doi.org/10.1186/1471-244X-12-89.

[8] de Araújo GS, Pereira SM, dos Santos DN, Marinho JM, Rodrigues LC, Barreto ML. Common mental disorders associated with tuberculosis: A matched case-control study. PLoS ONE 2014. https://doi.org/10.1371/journal.pone.0099551.

[9] Mcquistlon L, Susser E. Tuberculosis infection among people with severe mental illness. Psychiatric Services 1997;48:833–5. https://doi.org/10.1176/ps.48.6.833.

[10] Oh KH, Choi H, Kim EJ, Kim HJ, Cho SI. Depression & risk of tuberculosis: A nationwide populationbased cohort study. International Journal of Tuberculosis and Lung Disease 2017;21:804–9. https://doi.org/10.5588/ijtld.17.0038.

[11] Cheng KC, Liao KF, Lin CL, Lai SW. Increased risk of pulmonary tuberculosis in patients with depression: A cohort study in Taiwan. Frontiers in Psychiatry 2017;8. https://doi.org/10.3389/fpsyt.2017.00235.

[12] Pachi A, Bratis D, Moussas G, Tselebis A. Psychiatric Morbidity and Other Factors Affecting Treatment Adherence in Pulmonary Tuberculosis Patients. Tuberculosis Research and Treatment 2013. https://doi.org/10.1155/2013/489865.

[13] Duarte EC, Bierrenbach AL, da Silva JB, Tauil PL, de Fátima Duarte E. Factors associated with deaths among pulmonary tuberculosis patients: A case control study with secondary data. Journal of Epidemiology and Community Health 2009;63:233–8. https://doi.org/10.1136/jech.2008.078972.

[14] Martens WHJ. A review of physical and mental health in homeless persons. Public Health Reviews 2001.

[15] Oeltmann JE, Kammerer JS, Pevzner ES, Moonan PK. Tuberculosis and Substance Abuse in the United States, 1997-2006. Archives of Internal Medicine 2009. https://doi.org/10.1001/archinternmed.2008.535.

For peer review only

[16] Simon GE, von Korff M, Lin E. Clinical and functional outcomes of depression treatment in patients with and without chronic medical illness. Psychological Medicine 2005;35. https://doi.org/10.1017/S0033291704003071.

[17] Ambaw F, Mayston R, Hanlon C, Medhin G, Alem A. Untreated depression and tuberculosis treatment outcomes, quality of life and disability, Ethiopia. Bull World Health Organ 2018. https://doi.org/10.2471/BLT.17.192658.

[18] Sweetland AC, Kritski A, Oquendo MA, Sublette ME, Norcini Pala A, Silva LRB, et al. Addressing the tuberculosis–depression syndemic to end the tuberculosis epidemic. The International Journal of Tuberculosis and Lung Disease 2017. https://doi.org/10.5588/ijtld.16.0584.

[19] Lackey B, Seas C, van der Stuyft P, Otero L. Patient characteristics associated with tuberculosis treatment default: A cohort study in a high-incidence area of Lima, Peru. PLoS ONE 2015;10. https://doi.org/10.1371/journal.pone.0128541.

[20] Imtiaz S, Shield KD, Roerecke M, Samokhvalov A v., Lönnroth K, Rehm J. Alcohol consumption as a risk factor for tuberculosis: Meta-analyses and burden of disease. European Respiratory Journal 2017;50. https://doi.org/10.1183/13993003.00216-2017.

[21] Shyamala KK, Sharadha Naveen R, Khatri B. Depression: a neglected comorbidity in patients with tuberculosis. J Assoc Physicians India 2018;66:18–21.

[22] Acha J, Sweetland A, Guerra D, Chalco K, Castillo H, Palacios E. Psychosocial support groups for patients with multidrug-resistant tuberculosis: Five years of experience. Global Public Health 2007;2:404–17. https://doi.org/10.1080/17441690701191610.

[23] Walker IF, Baral SC, Wei X, Huque R, Khan A, Walley J, et al. Multidrug-resistant tuberculosis treatment programmes insufficiently consider comorbid mental disorders.

For peer review only

The International Journal of Tuberculosis and Lung Disease 2017. https://doi.org/10.5588/ijtld.17.0135.

[24] Sheehan D v., Lecrubier Y, Sheehan KH, Amorim P, Janavs J, Weiller E, et al. The Mini-International Neuropsychiatric Interview (M.I.N.I.): The development and validation of a structured diagnostic psychiatric interview for DSM-IV and ICD-10. Journal of Clinical Psychiatry, vol. 59, 1998.

[25] Lecrubier Y, Sheehan D v., Weiller E, Amorim P, Bonora I, Sheehan KH, et al. The Mini International Neuropsychiatric Interview (MINI). A short diagnostic structured interview: Reliability and validity according to the CIDI. European Psychiatry 1997. https://doi.org/10.1016/S0924-9338(97)83296-8.

[26] Sheehan D v., Lecrubier Y, Sheehan KH, Janavs J, Weiller E, Keskiner A, et al. The validity of the Mini International Neuropsychiatric Interview (MINI) according to the SCID-P and its reliability. European Psychiatry 1997. https://doi.org/10.1016/S0924-9338(97)83297-X.

[27] Otsubo T, Tanaka K, Koda R, Shinoda J, Sano N, Tanaka S, et al. Reliability and validity of Japanese version of the Mini-International Neuropsychiatric Interview. Psychiatry and Clinical Neurosciences 2005;59. https://doi.org/10.1111/j.1440-1819.2005.01408.x.

[28] Sheehan D v., Sheehan KH, Shytle RD, Janavs J, Bannon Y, Rogers JE, et al. Reliability and validity of the mini international neuropsychiatric interview for children and adolescents (MINI-KID). Journal of Clinical Psychiatry 2010;71. https://doi.org/10.4088/JCP.09m05305whi.

[29] Shittu RO, Issa BA, Olanrewaju GT, Mahmoud AO, Odeigah LO, Salami AK, et al. Prevalence and correlates of depressive disorders among people living with

For peer review only

HIV/AIDS, in north central Nigeria. Journal of AIDS and Clinical Research 2013;4. https://doi.org/10.4172/2155-6113.1000251.

[30] Wang XB, Li XL, Zhang Q, Zhang J, Chen HY, Xu WY, et al. A survey of anxiety and depressive symptoms in pulmonary tuberculosis patients with and without tracheobronchial tuberculosis. Frontiers in Psychiatry 2018;9:1–10. https://doi.org/10.3389/fpsyt.2018.00308.

[31] Aamir S, Aisha. Co-morbid anxiety and depression among pulmonary tuberculosis patients. Journal of the College of Physicians and Surgeons Pakistan 2010;20:703–4. https://doi.org/10.2010/JCPSP.703704.

[32] Naidoo P, Mwaba K. Helplessness, depression, and social support among people being treated for tuberculosis in South Africa. Social Behavior and Personality 2010;38. https://doi.org/10.2224/sbp.2010.38.10.1323.

[33] Theron G, Peter J, Zijenah L, Chanda D, Mangu C, Clowes P, et al. Psychological distress and its relationship with non-adherence to TB treatment: A multicentre study. BMC Infect Dis 2015;15:253. https://doi.org/10.1186/s12879-015-0964-2.

[34] Xu M, Markström U, Lyu J, Xu L. Survey on tuberculosis patients in rural areas in China: Tracing the role of stigma in psychological distress. International Journal of Environmental Research and Public Health 2017;14. https://doi.org/10.3390/ijerph14101171.

[35] Chen X, Wu R, Xu J, Wang J, Gao M, Chen Y, et al. Prevalence and associated factors of psychological distress in tuberculosis patients in Northeast China: a cross-sectional study. BMC Infectious Diseases 2021;21. https://doi.org/10.1186/s12879-021-06284-4.

For peer review only

[36] Duko B, Bedaso A, Ayano G. The prevalence of depression among patients with tuberculosis: A systematic review and meta-analysis. Annals of General Psychiatry 2020;19. https://doi.org/10.1186/s12991-020-00281-8.

[37] Schadé A, van Grootheest G, Smit JH. HIV-infected mental health patients: Characteristics and comparison with HIV-infected patients from the general population and non-infected mental health patients. BMC Psychiatry 2013;13. https://doi.org/10.1186/1471-244X-13-35.

[38] Remien RH, Stirratt MJ, Nguyen N, Robbins RN, Pala AN, Mellins CA. Mental health and HIV/AIDS: The need for an integrated response. AIDS 2019;33. https://doi.org/10.1097/QAD.0000000000002227.

[39] Gooden TE, Gardner M, Wang J, Chandan JS, Beane A, Haniffa R, et al. The risk of mental illness in people living with HIV in the UK: a propensity score-matched cohort study. The Lancet HIV 2022;9. https://doi.org/10.1016/S2352-3018(21)00319-2.

[40] Too EK, Abubakar A, Nasambu C, Koot HM, Cuijpers P, Newton CRJC, et al. Prevalence and factors associated with common mental disorders in young people living with HIV in sub-Saharan Africa: a systematic review. J Int AIDS Soc 2021;24. https://doi.org/10.1002/jia2.25705.

[41] Nyongesa MK, Mwangi P, Kinuthia M, Hassan AS, Koot HM, Cuijpers P, et al. Prevalence, risk and protective indicators of common mental disorders among young people living with HIV compared to their uninfected peers from the Kenyan coast: a cross-sectional study. BMC Psychiatry 2021;21. https://doi.org/10.1186/s12888-021-03079-4.

[42] Breuer E, Myer L, Struthers H, Joska JA. HIV/AIDS and mental health research in sub-Saharan Africa: A systematic review. African Journal of AIDS Research 2011;10. https://doi.org/10.2989/16085906.2011.593373.

For peer review only

[43] Zewdu S. Common Mental Disorder among HIV Infected Individuals at Comprehensive HIV Care and Treatment Clinic of Debre Markos Referral Hospital, Ethiopia. Journal of AIDS & Clinical Research 2015;06. https://doi.org/10.4172/2155-6113.1000420.

[44] Lampe FC. Increased risk of mental illness in people with HIV. The Lancet HIV 2022;9. https://doi.org/10.1016/S2352-3018(22)00034-0.

[45] Myer L, Smit J, Roux L le, Parker S, Stein DJ, Seedat S. Common mental disorders among HIV-infected individuals in South Africa: Prevalence, predictors, and validation of brief psychiatric rating scales. AIDS Patient Care and STDs 2008;22. https://doi.org/10.1089/apc.2007.0102.

[46] Tesfaye SH, Bune GT. Generalized psychological distress among HIV-infected patients enrolled in antiretroviral treatment in Dilla University Hospital, Gedeo zone, Ethiopia. Global Health Action 2014;7. https://doi.org/10.3402/gha.v7.23882.

[47] Morrison SD, Banushi VH, Sarnquist C, Gashi VH, Osterberg L, Maldonado Y, et al. Levels of self-reported depression and anxiety among HIV-positive patients in Albania: A cross-sectional study. Croatian Medical Journal 2011;52. https://doi.org/10.3325/cmj.2011.52.622.

[48] Evangeli M. Mental health and substance use in HIV-infected adolescents. Current Opinion in HIV and AIDS 2018;13. https://doi.org/10.1097/COH.0000000000000451.

[49] Fawzi MCS, Ng L, Kanyanganzi F, Kirk C, Bizimana J, Cyamatare F, et al. Mental health and antiretroviral adherence among youth living with HIV in Rwanda. Pediatrics 2016;138. https://doi.org/10.1542/peds.2015-3235.

[50] Mutumba M, Musiime V, Lepkwoski JM, Harper GW, Snow RC, Resnicow K, et al. Examining the relationship between psychological distress and adherence to anti-retroviral therapy among Ugandan adolescents living with HIV. AIDS Care -

For peer review only

Psychological and Socio-Medical Aspects of AIDS/HIV 2016;28. https://doi.org/10.1080/09540121.2015.1131966.

[51] Dow DE, Turner EL, Shayo AM, Mmbaga B, Cunningham CK, O'Donnell K. Evaluating mental health difficulties and associated outcomes among HIV-positive adolescents in Tanzania. AIDS Care - Psychological and Socio-Medical Aspects of AIDS/HIV 2016;28. https://doi.org/10.1080/09540121.2016.1139043.

[52] Olashore AA, Paruk S, Akanni OO, Tomita A, Chiliza B. Psychiatric Disorders in Adolescents Living with HIV and Association with Antiretroviral Therapy Adherence in Sub-Saharan Africa: A Systematic Review and Meta-analysis. AIDS and Behavior 2021;25. https://doi.org/10.1007/s10461-020-03100-z.

[53] Woimo TT, Yimer WK, Bati T, Gesesew HA. The prevalence and factors associated for anti-tuberculosis treatment non-adherence among pulmonary tuberculosis patients in public health care facilities in South Ethiopia: a cross-sectional study. BMC Public Health 2017;17. https://doi.org/10.1186/s12889-017-4188-9.

[54] Tesfahuneygn G, Medhin G, Legesse M. Adherence to Anti-tuberculosis treatment and treatment outcomes among tuberculosis patients in Alamata District, northeast Ethiopia. BMC Research Notes 2015;8. https://doi.org/10.1186/s13104-015-1452-x.

[55] Ifebunandu NA, Ukwaja KN. Tuberculosis treatment default in a large tertiary care hospital in urban Nigeria: Prevalence, trend, timing and predictors. Journal of Infection and Public Health 2012;5. https://doi.org/10.1016/j.jiph.2012.06.002.

[56] Addis Z, Birhan W, Alemu A, Mulu A, Ayal G, Negash H. Treatment Outcome of Tuberculosis Patients in Azezo Health Center, North West Ethiopia. International Journal of Biomedical and Advance Research 2013;4. https://doi.org/10.7439/ijbar.v4i3.300.

For peer review only

[57] Tekle B, Mariam DH, Ali A. Defaulting from DOTS and its determinants in three districts of Arsi Zone in Ethiopia. International Journal of Tuberculosis and Lung Disease 2002;6.

[58] Muture BN, Keraka MN, Kimuu PK, Kabiru EW, Ombeka VO, Oguya F. Factors associated with default from treatment among tuberculosis patients in nairobi province, Kenya: A case control study. BMC Public Health 2011;11. https://doi.org/10.1186/1471-2458-11-696.

[59] Lee LY, Tung HH, Chen SC, Fu CH. Perceived stigma and depression in initially diagnosed pulmonary tuberculosis patients. Journal of Clinical Nursing 2017;26. https://doi.org/10.1111/jocn.13837.

[60] Naidoo P, Peltzer K, Louw J, Matseke G, McHunu G, Tutshana B. Predictors of tuberculosis (TB) and antiretroviral (ARV) medication non-adherence in public primary care patients in South Africa: A cross sectional study. BMC Public Health 2013;13. https://doi.org/10.1186/1471-2458-13-396.

[61] Underner M, Perriot J, Peiffer G, Meurice J-C, Dautzenberg B. Smoking and adherence to anti-tuberculosis treatment. REVUE DES MALADIES RESPIRATOIRES 2016;33.

[62] Soboka M, Tesfaye M, Adorjan K, Krahl W, Tesfaye E, Yitayih Y, et al. Substance use disorders and adherence to antituberculosis medications in Southwest Ethiopia: A prospective cohort study. BMJ Open 2021;11. https://doi.org/10.1136/bmjopen-2020-043050.

[63] Shin SS, Mathew TA, Yanova G v., Fitzmaurice GM, Livchits V, Yanov SA, et al. Alcohol consumption among men and women with tuberculosis in Tomsk, Russia. Central European Journal of Public Health 2010;18. https://doi.org/10.21101/cejph.a3590.

For peer review only

[64] Shargie EB, Lindtjørn B. Determinants of treatment adherence among smear-positive pulmonary tuberculosis patients in Southern Ethiopia. PLoS Medicine 2007;4. https://doi.org/10.1371/journal.pmed.0040037.

[65] Amuha MG, Kutyabami P, Kitutu FE, Odoi-Adome R, Kalyango JN. Non-adherence to anti-TB drugs among TB/HIV co-infected patients in Mbarara Hospital Uganda: prevalence and associated factors. Afr Health Sci 2009;9 Suppl 1.

[66] Husain MO, Dearman SP, Chaudhry IB, Rizvi N, Waheed W. Clinical Practice and Epidemiology The relationship between anxiety , depression and illness perception in tberculosis patients in Pakistan. Clinical Practice and Epidemiology in Mental Health 2008;5.

[67] Janse Van Rensburg A, Dube A, Curran R, Ambaw F, Murdoch J, Bachmann M, et al. Comorbidities between tuberculosis and common mental disorders: A scoping review of epidemiological patterns and person-centred care interventions from low-to-middle income and BRICS countries. Infectious Diseases of Poverty 2020;9. https://doi.org/10.1186/s40249-019-0619-4.

[68] Suhadev M, Thomas BE, Raja Sakthivel M, Murugesan P, Chandrasekaran V, Charles N, et al. Alcohol use disorders (AUD) among tuberculosis patients: A study from Chennai, South India. PLoS ONE 2011;6. https://doi.org/10.1371/journal.pone.0019485.

[69] Thapa P, Kamath R, Shetty BK, Monteiro A, Sekaran VC. Prevalence and Associated Factors of Alcoholism among Tuberculosis Patients in Udupi Taluk, Karnataka, India: A Cross Sectional Study. J Nepal Health Res Counc 2014;12.

[70] Peltzer K, Louw J, Mchunu G, Naidoo P, Matseke G, Tutshana B. Hazardous and harmful alcohol use and associated factors in tuberculosis public primary care patients

For peer review only

in South Africa. International Journal of Environmental Research and Public Health 2012;9. https://doi.org/10.3390/ijerph9093245.

[71] Necho M, Tsehay M, Seid M, Zenebe Y, Belete A, Gelaye H, et al. Prevalence and associated factors for alcohol use disorder among tuberculosis patients: a systematic review and meta-analysis study. Substance Abuse: Treatment, Prevention, and Policy 2021;16. https://doi.org/10.1186/s13011-020-00335-w.

[72] Veerakumar AM, Sahu SK, Sarkar S, Kattimani S, Govindarajan S. Alcohol use disorders among pulmonary tuberculosis patients under RNTCP in urban Pondicherry, India. Indian Journal of Tuberculosis 2015;62. https://doi.org/10.1016/j.ijtb.2015.07.017.

[73] O'Connell R, Chishinga N, Kinyanda E, Patel V, Ayles H, Weiss HA, et al. Prevalence and Correlates of Alcohol Dependence Disorder among TB and HIV Infected Patients in Zambia. PLoS ONE 2013;8. https://doi.org/10.1371/journal.pone.0074406.

[74] Taquet M, Geddes JR, Husain M, Luciano S, Harrison PJ. 6-month neurological and psychiatric outcomes in 236 379 survivors of COVID-19: a retrospective cohort study using electronic health records. The Lancet Psychiatry 2021;8. https://doi.org/10.1016/S2215-0366(21)00084-5.

[75] Lebina L, Dube M, Hlongwane K, Brahmbatt H, Lala SG, Reubenson G, et al. Trends in paediatric tuberculosis diagnoses in two south african hospitals early in the covid-19 pandemic. South African Medical Journal 2020;110. https://doi.org/10.7196/SAMJ.2020.v110i12.15386.

[76] Kwak N, Hwang SS, Yima AJ. Effect of COVID-19 on Tuberculosis Notification, South Korea. Emerging Infectious Diseases 2020;26. https://doi.org/10.3201/EID2610.202782.

For peer review only

[77] Hogan AB, Jewell BL, Sherrard-Smith E, Vesga JF, Watson OJ, Whittaker C, et al. Potential impact of the COVID-19 pandemic on HIV, tuberculosis, and malaria in low-income and middle-income countries: a modelling study. The Lancet Global Health 2020;8. https://doi.org/10.1016/S2214-109X(20)30288-6.

For peer review only

Tables

	Number	Percentage
**Sex**		
Male	122	61.9%
Female	75	38.1%
**Age**		
Adolescents (<19 years)	4	2%
Adults (19 – 44)	149	76%
Middle age (45 – 64)	33	16.8%
Aged+ (65+)	10	5.1%
**Relationship status**		
Single	139	70.6%
Married	44	22.3%
Divorced/separated	14	7.1%
**Level of education**		
Primary school	33	16.8%
High school	112	56.9%
Tertiary education	52	26.4%
**Employment status**		
Employed	84	42.6%
Unemployed	113	57.4%
Receiving state grant/pension	26	13.2%
**Monthly income[a]**		
Less than R2 500	127	64.5%
More than R2 500	70	35.5%
**HIV status**		
Negative	62	31.5%
Positive	128	64.97%
Unknown	7	3.6%
**Taking ART**		
Yes	99	77.3%
No	29	22.6%
**Disease classification**		
Pulmonary	153	77.7%

For peer review only

Extrapulmonary	44	22.3%
**Phase of TB treatment**		
Active	102	51.8%
Continuation	95	48.2%
**Abbreviations**: ART - Antiretroviral therapy; TB - Tuberculosis. [a] - SA Poverty line is R1 220		

For peer review only

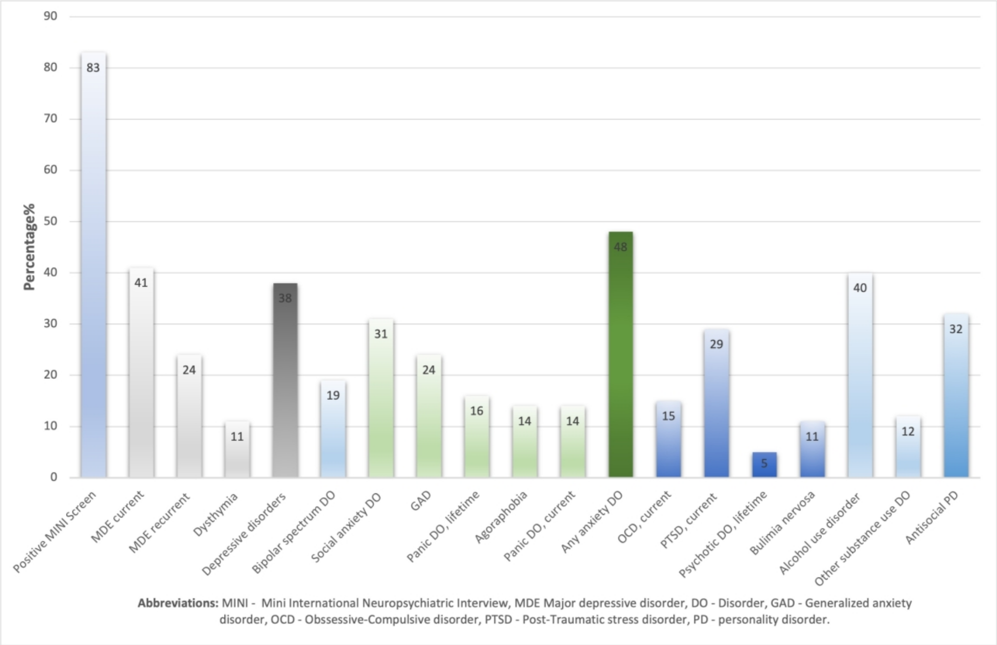

Figure 1: Common mental disorders in TB patients (N =197).

239x155mm (330 x 330 DPI)

BMJ Open

The Co-Morbidity of Mental Ill-Health in Tuberculosis Patients under Treatment in a Rural Province of South Africa – A cross-sectional survey.

Journal:	*BMJ Open*
Manuscript ID	bmjopen-2021-058013.R2
Article Type:	Original research
Date Submitted by the Author:	24-Aug-2022
Complete List of Authors:	Thungana, Yanga; Walter Sisulu University, Department of Psychiatry and Behavioural Sciences<br>Wilkinson, Robert; Francis Crick Institute, Pathology; Imperial College London<br>Zingela, Zukiswa; Nelson Mandela University, Executive Dean's Office, Faculty of Health Sciences
**Primary Subject Heading**:	Mental health
Secondary Subject Heading:	Infectious diseases, Mental health, Public health
Keywords:	Tuberculosis < INFECTIOUS DISEASES, Substance misuse < PSYCHIATRY, Adult psychiatry < PSYCHIATRY, PUBLIC HEALTH

SCHOLARONE™
Manuscripts

I, the Submitting Author has the right to grant and does grant on behalf of all authors of the Work (as defined in the below author licence), an exclusive licence and/or a non-exclusive licence for contributions from authors who are: i) UK Crown employees; ii) where BMJ has agreed a CC-BY licence shall apply, and/or iii) in accordance with the terms applicable for US Federal Government officers or employees acting as part of their official duties; on a worldwide, perpetual, irrevocable, royalty-free basis to BMJ Publishing Group Ltd ("BMJ") its licensees and where the relevant Journal is co-owned by BMJ to the co-owners of the Journal, to publish the Work in this journal and any other BMJ products and to exploit all rights, as set out in our licence.

The Submitting Author accepts and understands that any supply made under these terms is made by BMJ to the Submitting Author unless you are acting as an employee on behalf of your employer or a postgraduate student of an affiliated institution which is paying any applicable article publishing charge ("APC") for Open Access articles. Where the Submitting Author wishes to make the Work available on an Open Access basis (and intends to pay the relevant APC), the terms of reuse of such Open Access shall be governed by a Creative Commons licence – details of these licences and which Creative Commons licence will apply to this Work are set out in our licence referred to above.

Other than as permitted in any relevant BMJ Author's Self Archiving Policies, I confirm this Work has not been accepted for publication elsewhere, is not being considered for publication elsewhere and does not duplicate material already published. I confirm all authors consent to publication of this Work and authorise the granting of this licence.

For peer review only

The Co-Morbidity of Mental Ill-Health in Tuberculosis Patients under Treatment in a Rural Province of South Africa – A cross-sectional survey.

Yanga Thungana*[1], Robert John Wilkinson[2,3,4], Zukiswa Zingela[1]

1. Department of Psychiatry and Behavioural Sciences, Walter Sisulu University, Mthatha, South Africa
2. Wellcome Center for Infectious Diseases Research in Africa, Institute of Infect. Disease and Mol. Med and Dept. med. Univ. Cape Town, South Africa
3. Department of Infectious Disease, Imperial College, London, W12 ONN, UK
4. The Francis Crick Institute, London, NW1 IAT, UK

Correspondence address:

Yanga Thungana*

Department of psychiatry, Walter Sisulu University

Sisson street, Fort Gale, Mthatha, 5100

ythungana@wsu.ac.za

For peer review only

Abstract

Objectives: Tuberculosis (TB) remains prevalent despite the availability of effective anti-TB medications, and accumulating evidence suggests a high rate of mental disorders in people with TB. This is because TB and psychiatric disorders share several risk factors, such as poverty, homelessness, and substance use disorder. Moreover, psychiatric comorbidities in TB patients are associated with poor treatment outcomes. This study explored the psychiatric comorbidity and clinical correlates in individuals receiving TB treatment.

Design*:* A cross-sectional survey over ten months.

Setting: Two primary care clinics at King Sabata Dalindyebo District (KSD), Mthatha, Eastern Cape, South Africa.

Participants: Patients receiving TB treatment in the two clinics.

Interventions: The Mini-International Neuropsychiatric Interview was used to screen for psychiatric disorders.

Primary and Secondary Outcome Measures: Rates of mental disorders in TB patients over a ten-month period. Variation in rates by sex, employment status, and HIV comorbidity.

Results: In a sample of 197 participants, most patients were male (62%) and screened positive for a mental disorder (82%) with anxiety (48%), depression (38%), and substance use disorders (43%) being the most common psychiatric conditions. On average, individuals had 4 (SD 2) mental disorders. Females had higher rates of depression ($p = 0.005$) and nonadherence to TB treatment ($p = 0.003$), and alcohol use disorder was more common in males ($p< 0.001$) and in those nonadherent to TB treatment. Additionally, low education levels and unemployment were associated with depressive and anxiety disorders ($p < 0.05$).

Conclusions: Mental disorders are common in patients with tuberculosis, and mental health services need to be integrated into the management of patients with TB. Factors linked to mental disorders in this cohort, such as low education, gender, and unemployment, may be

For peer review only

useful for compiling a risk profile to help identify those with TB who may require more intensive support for their mental health.

Strengths and limitations of this study

- Using a structured clinical diagnostic interview improved consistency in the individual assessments.
- The MINI covers a broad range of mental disorders, and the most common psychiatric disorders are included in the scale.
- As a cross-sectional study, causal inferences could not be drawn between variables
- The inclusion of only rural participants and a small sample could limit the generalizability of study findings

For peer review only

Despite the availability of effective anti-tuberculosis drugs, tuberculosis (TB) remains one of the top ten causes of death worldwide, and it is among the leading causes of death from a single infectious agent. [1] South Africa is among the top eight countries that account for two-thirds of the global TB infections. [1] Additionally, in people infected with TB, there are high rates of mental disorders such as depression and anxiety disorders. [2–5] Depression is one of the most common comorbid psychiatric disorders in people with TB and is estimated to be 3-times higher than in patients without TB. [6–8] Conversely, people with psychiatric disorders may be at an increased risk of developing TB as well. [9–11]

There are several postulated reasons for the increased prevalence of mental disorders in people with TB, including shared risk factors such as homelessness, HIV/AIDS, substance use, stigma, malnutrition, and poor socioeconomic status. [12–15] Psychiatric comorbidity in TB is associated with poor treatment outcomes, disability, treatment failure, and poor quality of life due to poor health-seeking behaviours and challenges with adherence to medication in this cohort of patients. [16–18] Substance use, especially alcohol use, is prevalent in patients with tuberculosis and is thought to increase TB incidence, TB mortality, delayed presentation to health facilities, and poor adherence to TB treatment. [19–21] Furthermore, there is evidence that treating comorbid psychiatric disorders can improve TB outcomes. [22]

Although the relationship between TB and mental disorders is described, there is often a lack of integration of mental health services in the management of patients with TB. [23] Additionally, in South Africa, limited studies have explored in detail the prevalence of psychiatric disorders in people with TB. The value of this study is in the use of a different psychiatry questionnaire (Mini-International Neuropsychiatric Interview) that is more extensive and evaluates many more different psychiatric disorders compared to previous

For peer review only

studies evaluating mental disorders in TB patients. Also, unique to our study is that the MINI is observer administered compared to the commonly used self-report questionnaires. Therefore, we sought to investigate the point prevalence of comorbid mental disorders and its clinical correlates in patients receiving TB treatment in two district clinics in a rural area in the Eastern Cape, South Africa.

METHODS

Sample and procedure

A cross-sectional study was conducted among TB patients attending two primary care clinics (Mthatha Gateway and Stanford Terrace) at King Sabata Dalindyebo District (KSD), Mthatha, Eastern Cape, South Africa. Both clinics offer general medical care, including TB screening and the management of patients with TB. The study was conducted over a ten-month period, from September 2020 to June 2021. Participants included in the study were all patients currently diagnosed and receiving TB treatment who attended the two clinics during the study period. In these clinics, each morning, a nurse holds a short meeting with patients attending the clinic on the day to triage and guide them on accessing services. It is in these morning meetings that the nurse would identify all patients coming to collect tuberculosis treatment and would inform the research assistant (RA), who then approached each patient individually. Due to the coronavirus (Covid-19) pandemic and applicable South African lockdown restrictions at the time, the total number of patients attending clinics was significantly reduced.

For peer review only

The RA would explain the study to each patient individually in their language, obtain written informed consent, and assure them that their personally identifying information would be kept confidential at all times. Excluded from the study were participants under 18 years of age and those who could not give informed consent. A total of 197 participants were approached by the RA; all provided written consent and thus were included in the study. For our study, a mental disorder, also referred to as a psychiatric disorder, is a condition with a clinically significant disturbance of an individual's emotions, cognition, or behaviour associated with personal distress or impairment in adaptive function. And would include all the conditions recognized in the DSM-5. Psychiatric comorbidity or comorbid psychiatric disorder in our study refers to diagnosing a mental disorder in a patient with current tuberculosis.

Data collection

The RA had prior experience in mental health research and was trained on using the MINI over ten working days by a psychiatrist. The training was held for 90 minutes each day regarding how to use the different modules of the MINI and an explanation of the various psychiatric terms used in the MINI. The last two sessions were role-playing interviews to assess the RA's level of understanding of the MINI. Lastly, the RA received supervision throughout the period of data collection from the lead author, who is a qualified specialist psychiatrist and a neuropsychiatrist fellow at the time of the study.

The English version of the MINI was administered in English to all study participants. The RA was instructed to read the questions slowly, repeat them as necessary, and encourage the patients to seek clarity on any question that is not entirely clear to them. The RA was

For peer review only

instructed to schedule at least an hour to accommodate those slow in answering the numerous questions of the MINI. Lastly, when a patient did not understand a question, the RA was allowed to explain it in the patient's primary language.

Measures

A data capture sheet was used to collect sociodemographic details, past psychiatric history, medical history including HIV status, and adherence to TB treatment. Data on whether a participant had defaulted on their current TB treatment were also sought from the patient, although their responses were not verified through other means. The patients were asked whether they had stopped taking the TB treatment for at least 7 consecutive days.

The Mini-International Neuropsychiatric Interview (MINI) was developed as a brief, structured diagnostic interview used as a measure to diagnose several psychiatric disorders. [24] It has demonstrated good psychometric properties as a tool to screen for several psychiatric disorders. [24–26] For instance, the specificity of the MINI is at least good to excellent (0.72 – 1.0) for the diverse conditions screened is used to evaluate. [25,27,28] Although the MINI contains over 100 questions and screens for 17 mental disorders, it can be administered over a short period – approximately 20 – 30 minutes. The MINI is organised into diagnostic modules. For nearly all the modules, two to four screening questions are used to exclude a diagnosis if answered negatively. Conversely, positive responses to the screening questions lead to further inquiry about other diagnostic criteria. The MINI modules include a major depressive disorder (current and recurrent), dysthymia, manic & hypomanic episodes (current and past), panic disorder (current and lifetime), agoraphobia, social phobia, obsessive-compulsive disorder, post-traumatic stress disorder, alcohol abuse & dependence,

For peer review only

non-alcohol substance use & dependence, psychotic disorders (current and lifetime), anorexia nervosa, bulimia nervosa, generalized anxiety disorder, and antisocial personality disorder. We used all the MINI modules in our study.

Patient and public involvement

There was no involvement of patients in the design, recruitment, and conduct of the study. The study results will not be distributed to the individual participants, but the published paper will be available in the participating clinics.

Data analytical plan

Data were analysed using STATA/SE "(version 16.1 for Mac)". Descriptive statistics such as frequencies and proportions for categorical variables, median and interquartile range (IQR), or mean and standard deviation for continuous variables depending on the data skewness were utilised to characterise the sample. Inferential statistics were applied to evaluate the association between different variables. Firstly, to assess associations between categorical variables, we performed either the Pearson's chi-square test or Fischer's exact test, depending on the number of scored items under each variable. Secondly, for the continuous variables, we first assessed for normality using the Skewness and Kurtosis test (*sktest*) with a $p > 0.05$ indicating normal distribution. Therefore, due to the skewness of our data, we used either the Wilcoxon rank-sum test or the Kruskal-Wallis test to evaluate associations between numerical and categorical variables. The significance level was set at $P < 0.05$ for all the bi-variate hypothesis testing.

For peer review only

RESULTS

Most of the 197 participants were men (61.9%, n = 122), and the median age was 34 years (IQR 28-42) of age (**Table 1**). Most of the participants were single (70.6%, n = 139), had at least secondary education (56.9%, n= 112), and were unemployed (57.4%, n = 113). We did not find any statistically significant associations between age and the different psychiatric disorders. For instance, the adult group (44%, n = 66) had the highest proportion of participants with an alcohol use disorder compared to other age groups, but it was not statistically significant (p = 0.055). Unemployed participants were more likely to have an anxiety disorder (p = 0.03), depressive disorder (p < 0.001), and obsessive-compulsive disorder (OCD) (p = 0.006). Moreover, individuals earning less than R2500.00 a month had a higher proportion of depressive disorders (74.7%, p < 0.001), social phobia (75.4%, p = 0.03), and agoraphobia (85.7%, p = 0.01) than those earning more than R2500.00. Lower levels of education were associated with recurrent depressive disorder (P = 0.001), lifetime panic disorder (p = 0.02), ongoing generalized anxiety disorder (p = 0.02), and current OCD (p = 0.03). But social anxiety disorder was more common in those with at least a high school education (p = 0.02).

Table 1. Sociodemographic details of the participants (N=197)

	Number	Percentage
Sex		
Male	122	61.9%
Female	75	38.1%
Age		
Adolescents (<19 years)	4	2%
Adults (19 – 44)	149	76%
Middle age (45 – 64)	33	16.8%

For peer review only

Aged+ (65+)	10	5.1%
**Relationship status**		
Single	139	70.6%
Married	44	22.3%
Divorced/separated	14	7.1%
**Level of education**		
Primary school	33	16.8%
High school	112	56.9%
Tertiary education	52	26.4%
**Employment status**		
Employed	84	42.6%
Unemployed	113	57.4%
Receiving state grant/pension	26	13.2%
**Monthly income[a]**		
Less than R2 500	127	64.5%
More than R2 500	70	35.5%
**HIV status**		
Negative	62	31.5%
Positive	128	64.97%
Unknown	7	3.6%
**Taking ART**		
Yes	99	77.3%
No	29	22.6%
**Disease classification**		
Pulmonary	153	77.7%
Extrapulmonary	44	22.3%
**Phase of TB treatment**		
Active	102	51.8%
Continuation	95	48.2%

Abbreviations: ART - Antiretroviral therapy; TB - Tuberculosis. [a] - SA Poverty line is R1 220

Medical history

For peer review only - http://bmjopen.bmj.com/site/about/guidelines.xhtml

The majority of the participants were HIV positive (65%, n = 128) (95% CI 58.0 - 71.4) with significantly higher proportion of males (78.4%, n = 58) having HIV than females (60.7, n = 71) participants (p = 0.01). Most participants with an HIV diagnosis were receiving antiretroviral therapy (77.3%, n = 99) (**Table 1**). Most HIV-positive (96.6%, n = 28) participants not on ART were still in the intensive phase of TB treatment. Moreover, most of the participants in the sample (77.7%, n = 153) had pulmonary TB, and more than half (51.8%, n = 102) were in the intensive phase of TB treatment. Only a minority of patients (9.1%, n = 18) reported non-adherence to their TB treatment. Most participants reporting non-adherence to TB treatment were HIV positive (77.8%, p = 0.4), still in the intensive phase (55.6%, p = 0.8) of TB treatment, unemployed (55.6%, p = 0.8), and assessed to have a mental disorder (88.9%, p = 0.4) but these did not reach statistical significance. However, being female (p = 0.003) and having an alcohol use disorder (p = 0.001) were significantly associated with non-adherence to TB treatment.

Mental disorders

Most of the participants (82.7%, n = 163) screened positive for at least one psychiatric disorder, excluding substance use disorders (***Fig 1***), and the proportion of males (82.1%, n = 101) with a positive screen was similar to that of female (84%, n = 63) participants. Furthermore, the HIV status of the participants was not associated with screening outcomes for psychiatric disorders, with those HIV negative almost screening equally positive for a psychiatric disorder as those who were HIV positive (80.7% HIV-negative vs. 85.2% HIV-positive; p = 0.4). There was a comparable proportion of individuals assessed to have a psychiatric diagnosis in those in the intensive phase (82.5%, n = 85) and a continuation phase (83.2%, n = 79) of TB treatment.

For peer review only

The most common psychiatric disorders were anxiety disorders (48.2%, n = 95), current major depressive episodes (40.6%, n = 80), unipolar depression (37.6%, n = 74), post-traumatic stress disorder (28.9%, n = 57), antisocial personality (23.9%, n = 47), and bipolar spectrum conditions (19.3%, n = 38). Approximately a quarter (25.7%, n = 19) of the patients with unipolar depression currently had suicidal ideation. The patients with a positive diagnosis of a mental disorder had an average of 3.5 (SD 2.3) disorders excluding substance use disorders. Also, patients tended to have more than one anxiety disorder with an average of 2 (SD 1) disorders per individual.

More participants (47%) in the intensive phase of TB treatment had a current depressive episode compared to those in the continuation phase (34%); however, this was not statistically significant ($p = 0.056$). Noteworthily, female participants were more likely to have a depressive disorder than male participants ($p = 0.005$). In contrast, males were more likely to screen positive for an antisocial personality disorder ($p < 0.001$).

Substance use disorders

A considerable number of the participants had a substance use disorder (43.2%, n = 85), with more participants having an alcohol use disorder (40.1%, n = 79) than those having a substance use disorder other than alcohol (12.2%, n = 24). However, most of the 24 participants (79.2%, n = 19) with other substance use disorders also had alcohol use disorder. Nevertheless, both alcohol use disorders ($p = 0.001$) and other substance use disorders ($p = 0.001$) were more common among male participants. In this sample, there was no significant association between HIV status and substance use disorders.

For peer review only

DISCUSSION

In this study, we examined the psychiatric comorbidity in outpatients receiving TB treatment. We found a high rate of mental disorders in patients with TB; depression, anxiety, and substance use disorders were the most common mental disorders. Additionally, those with mental disorders approximately had 4 (SD 2) lifetime psychiatric disorders, excluding substance use disorders. Also, alcohol use disorder was prevalent in our study participants and was significantly associated with the male sex and poor adherence to TB treatment. Female sex was associated with depressive disorders and nonadherence to treatment. Lastly, low levels of education and unemployment were associated with a higher rate of depressive, anxiety, and obsessive-compulsive disorders.

The prevalence of psychiatric disorders in patients with tuberculosis varies greatly among different studies from less than 20% to about 80% with a trend of higher rates in lower-middle-income countries (LMIC) [21,29–31]. In South Africa (SA), the prevalence of psychiatric comorbidity in TB patients is similar to other LMIC. For instance, the prevalence of depression is documented to be 64.3%, and PTSD at 29.6%.[6,32] But higher rates of psychological distress (81%) have been reported in SA.[6,7] In our study, we found an overall higher rate of mental disorders (approx. 83%), but the rates of PTSD and depression were similar and lower to previous studies, respectively.[6,32] The major variation in prevalence in our study could be due to several factors such as the assessment instrument used, a high HIV comorbidity, the sociodemographic factors, or that it is a true reflection of the high psychiatric comorbidity in this population. For instance, HIV comorbidity in patients with TB is associated with higher mental morbidity.[33] Also, South Africa is a middle-

For peer review only - http://bmjopen.bmj.com/site/about/guidelines.xhtml

income country with a very high TB burden; the personal socioeconomic consequences of TB infection might be amplified, thus leading to more pronounced mental disorders. [1,33] A case in point documented is that in TB patients, unemployment, poverty, and lower educational background are associated with higher psychiatric comorbidity, as was the case in our study. [6,34,35]

Furthermore, the MINI covers a broader number of conditions than scales used in other studies, and in patients with TB, mental disorders have been found to vary significantly depending on the psychiatric screening scale used. [36] Compared to previous studies that tended to assess broader categories of mental disturbance such as anxiety or psychological distress, we were able to screen for several individual psychiatric diagnoses. Thus, in addition to describing the presence of mental illness, we were able to assess the number of disorders experienced by each individual and we found a tendency for participants to have more than one psychiatric disorder with an average of approx. 4 (SD 2) disorders per individual, excluding substance use disorders.

In this study, we did not find a statistically significant relationship between HIV-positive individuals and an increased rate of mental disorders. But the finding of increased psychiatric disorders in PLWH has been described. [37–39] However, the rate of mental illness in PLWH varies significantly from as low as below 10% to as high as over 80%. [29,40–43] The wide range in the prevalence could be due to the different study designs, study settings, and measurement instruments used in different studies. Moreover, not all studies have found an increased rate of mental illness in PLWH. [44]

For peer review only

We found a slightly higher adherence rate to TB treatment (91%) in the current study. Previous studies have documented adherence rates between 70% and 89%. [45–48] The higher rates of treatment adherence in our study could be due to our methodology, as we solely relied on the patient's report and did investigate it further. Non-adherence to treatment in patients with TB can be due to several factors, including HIV seropositivity, substance use, distance to health facilities, male gender, running out of medication, medication factors, the continuation phase of chemotherapy, and stigma. [45–47] Additionally, psychiatric comorbidity has been described as another factor with a negative impact on compliance with TB treatment.[5,33,49,50] Also, substance use is associated with poor adherence to TB treatment,[50–52] as was the case in our study. Contrary to findings in the current study, previous studies have found better adherence to tuberculosis treatment among female participants.[47,53] Furthermore, the proportion of participants with psychiatric disorders was comparable between those in the intensive phase and continuation phase of chemotherapy. But the continuation phase of TB treatment is previously associated with a higher rate of mental illness.[48,54,55]

Our finding that anxiety and depressive disorders are the most common mental disorders in patients with TB is comparable to previous studies. [21,36,56] Moreover, as in our study, women with TB were more likely to suffer from depressive disorders than male patients.[33,36] It has been reported that psychiatric disorders are more common in the earlier phase of TB infection and rates decline as patients recover from TB infection.[21] Our study showed a similar trend with more patients in the intensive phase of treatment having a current major depressive episode than those in the continuation phase; however, the difference did not reach statistical significance.

For peer review only

Alcohol use disorder was common in our study sample, corroborating a finding in previous literature highlighting high rates of alcohol use in patients with TB.[57–60] In those already infected with TB, alcohol abuse is associated with a lack of cooperation, poor adherence, higher levels of depression, and mortality.[19,20] Similar to previous studies, alcohol use disorders were more common among the male participants in our study.[59,61] However, the prevalence of alcohol use disorder (40%) in our study was higher than previously reported and could be because most participants were males, single, unemployed, and had lower education levels.[57,60]

Strengths and limitations of this study

The use of the MINI, a well-established structured diagnostic interview scale that covers a broader number of mental disorders, adds to the study's strength. Furthermore, all patients with TB attending the two clinics were admitted to the study with no exclusion criteria. The study was conducted during the COVID-19 pandemic, which possibly affected clinic attendance and TB screening and could have contributed to the high prevalence of mental disorders.[62,63] Along with this, the small study sample could restrain the generalizability of the study results to other settings. Additionally, we did not conduct individual clinical assessments to confirm the diagnosis of the MINI, which could possibly increase the positive psychiatric diagnosis. Lastly, the MINI was administered in English to participants to whom English is not their primary language, which might have affected the accuracy of the patient's responses.

CONCLUSION

For peer review only

Our study showed an increased frequency and number of mental disorders among individuals receiving TB treatment. Moreover, as in previous studies, we found substance use disorders prevalent in TB patients. Thus, the study outcomes further highlight the need to integrate mental health services in managing patients with TB at the primary care level, including screening all new TB patients. There is a need for studies to evaluate whether brief interventions for substance use at the primary care level could improve adherence and recovery from tuberculosis.

ETHICS STATEMENTS

Patient consent for publication

Not required

Ethics approval

Ethical approval for this study was obtained from the Walter Sisulu University Human Research Committee (protocol number 119/2019) and the South African Department of Health (EC_202006_009). Each participant, before taking part in the study, gave a signed informed consent in their primary language of communication.

ACKNOWLEDGEMENTS

We would like to express our gratitude to our research assistant, Simthembile Lindani, who worked tirelessly and completed data collection despite the challenges of the COVID-19 pandemic. We would like to thank Editage (www.editage.com) for English language editing.

COMPETING INTERESTS

All authors have completed the ICMJE uniform disclosure form and declare no conflict of interest.

FUNDING

For peer review only

This research was Supported by the National Institutes of Health grant number (NIH U19AI115940). RJW is supported by the Francis Crick Institue which receives funding from Wellcome (FC0010218), Cancer Research UK (FC0010218), and the Medical Research Council (FC0010218). For the purpose of open access, the authors have applied a CC-BY public copyright to any author-accepted manuscript arising from this submission.

DATA SHARING STATEMENT

The data (deidentified participants dataset) can be available upon reasonable request from the corresponding author.

AUTHORS' CONTRIBUTIONS

YT, RJW, and ZZ were involved in the conception and design of the study. YT analysed the data, and YT wrote the manuscript. YT, RJW, and ZZ revised the manuscript. All authors provided their final approval for manuscript submission and agree to be accountable for all aspects of the work. YT, as the corresponding author, confirms that all authors meet the authorship criteria and that no others have been omitted.

Table 1: The sociodemographic details of the survey sample

Figure 1: A proportion of patients with mental disorders among tuberculosis patients (N = 197)

For peer review only

REFERENCES

[1] Global tuberculosis report. Geneva: World Health Organization; 2020. International Journal of Infectious Diseases 2021. https://doi.org/10.1016/j.ijid.2021.02.107.

[2] Mason PH, Sweetland AC, Fox GJ, Halovic S, Nguyen TA, Marks GB. Tuberculosis and mental health in the Asia-Pacific. Australasian Psychiatry 2016;24:553–5. https://doi.org/10.1177/1039856216649770.

[3] Koyanagi A, Vancampfort D, Carvalho AF, DeVylder JE, Haro JM, Pizzol D, et al. Depression comorbid with tuberculosis and its impact on health status: cross-sectional analysis of community-based data from 48 low- and middle-income countries. BMC Med 2017;15:209. https://doi.org/10.1186/s12916-017-0975-5.

[4] Doherty AM, Kelly J, McDonald C, O'Dywer AM, Keane J, Cooney J. A review of the interplay between tuberculosis and mental health. Gen Hosp Psychiatry 2013;35:398–406. https://doi.org/10.1016/j.genhosppsych.2013.03.018.

[5] Ruiz-Grosso P, Cachay R, de La Flor A, Schwalb A, Ugarte-Gil C. Association between tuberculosis and depression on negative outcomes of tuberculosis treatment: a systematic review and meta-analysis. PLoS ONE 2020;15:e0227472. https://doi.org/10.1371/journal.pone.0227472.

[6] Peltzer K, Naidoo P, Matseke G, Louw J, McHunu G, Tutshana B. Prevalence of post-traumatic stress symptoms and associated factors in tuberculosis (TB), TB retreatment and/or TB-HIV co-infected primary public health-care patients in three districts in South Africa. Psychol Health Med 2013;18:387–97. https://doi.org/10.1080/13548506.2012.726364.

[7] Peltzer K, Naidoo P, Matseke G, Louw J, Mchunu G, Tutshana B. Prevalence of psychological distress and associated factors in tuberculosis patients in public primary

For peer review only

care clinics in South Africa. BMC Psychiatry 2012;12:89. https://doi.org/10.1186/1471-244X-12-89.

[8] de Araújo GS, Pereira SM, dos Santos DN, Marinho JM, Rodrigues LC, Barreto ML. Common mental disorders associated with tuberculosis: a matched case-control study. PLoS ONE 2014;9:e99551. https://doi.org/10.1371/journal.pone.0099551.

[9] Mcquistlon L, Susser E. Tuberculosis infection among people with severe mental illness. Psychiatr Serv 1997;48:833–5. https://doi.org/10.1176/ps.48.6.833.

[10] Oh KH, Choi H, Kim EJ, Kim HJ, Cho SI. Depression and risk of tuberculosis: a nationwide populationbased cohort study. Inte J Tuberc Lung Dis 2017;21:804–9. https://doi.org/10.5588/ijtld.17.0038.

[11] Cheng KC, Liao KF, Lin CL, Lai SW. Increased risk of pulmonary tuberculosis in patients with depression: a cohort study in Taiwan. Frontiers in Psychiatry 2017;8:235. https://doi.org/10.3389/fpsyt.2017.00235.

[12] Pachi A, Bratis D, Moussas G, Tselebis A. Psychiatric morbidity and other factors affecting treatment adherence in pulmonary tuberculosis patients. Tuberculosis Research and Treatment 2013;2013:489865. https://doi.org/10.1155/2013/489865.

[13] Duarte EC, Bierrenbach AL, da Silva JB, Tauil PL, de Fátima Duarte E. Factors associated with deaths among pulmonary tuberculosis patients: a case control study with secondary data. Journal of Epidemiology and Community Health 2009;63:233–8. https://doi.org/10.1136/jech.2008.078972.

[14] Martens WHJ. A review of physical and mental health in homeless persons. Public Health Rev 2001;29:13–33.

[15] Oeltmann JE, Kammerer JS, Pevzner ES, Moonan PK. Tuberculosis and Substance Abuse in the United States, 1997-2006. Arch Intern Med 2009;169:189–97. https://doi.org/10.1001/archinternmed.2008.535.

For peer review only

[16] Simon GE, von Korff M, Lin E. Clinical and functional outcomes of depression treatment in patients with and without chronic medical illness. Psychol Med 2005;35:271–9. https://doi.org/10.1017/S0033291704003071.

[17] Ambaw F, Mayston R, Hanlon C, Medhin G, Alem A. Untreated depression and tuberculosis treatment outcomes, quality of life and disability, Ethiopia. Bull World Health Organ 2018;96:243–55. https://doi.org/10.2471/BLT.17.192658.

[18] Sweetland AC, Kritski A, Oquendo MA, Sublette ME, Norcini Pala A, Silva LRB, et al. Addressing the tuberculosis–depression syndemic to end the tuberculosis epidemic. Intern J Tuberc Lung Dis 2017;2:852–61. https://doi.org/10.5588/ijtld.16.0584.

[19] Lackey B, Seas C, van der Stuyft P, Otero L. Patient characteristics associated with tuberculosis treatment default: a cohort study in a high-incidence area of Lima, Peru. PLoS ONE 2015;10:e0128541. https://doi.org/10.1371/journal.pone.0128541.

[20] Imtiaz S, Shield KD, Roerecke M, Samokhvalov A v., Lönnroth K, Rehm J. Alcohol consumption as a risk factor for tuberculosis: meta-analyses and burden of disease. Eur Respir J 2017;50:1700216. https://doi.org/10.1183/13993003.00216-2017.

[21] Shyamala KK, Sharadha Naveen R, Khatri B. Depression: A neglected comorbidity in patients with tuberculosis. Journal of Association of Physicians of India 2018;66:18–21.

[22] Acha J, Sweetland A, Guerra D, Chalco K, Castillo H, Palacios E. Psychosocial support groups for patients with multidrug-resistant tuberculosis: five years of experience. Global Public Health 2007;2:404–17. https://doi.org/10.1080/17441690701191610.

[23] Walker IF, Baral SC, Wei X, Huque R, Khan A, Walley J, et al. Multidrug-resistant tuberculosis treatment programmes insufficiently consider comorbid mental disorders. Intern J Tuberc Lung Dis 2017;21:603–9. https://doi.org/10.5588/ijtld.17.0135.

For peer review only

[24] Sheehan D v., Lecrubier Y, Sheehan KH, Amorim P, Janavs J, Weiller E, et al. The Mini-International Neuropsychiatric Interview (M.I.N.I.): The development and validation of a structured diagnostic psychiatric interview for DSM-IV and ICD-10. J Clin Psychiatry, vol. 59, 1998, p. 22–33.

[25] Lecrubier Y, Sheehan D v., Weiller E, Amorim P, Bonora I, Sheehan KH, et al. The Mini International Neuropsychiatric Interview (MINI): a short diagnostic structured interview: Reliability and validity according to the CIDI. Eur Psychiatry 1997;12:224–31. https://doi.org/10.1016/S0924-9338(97)83296-8.

[26] Sheehan D v., Lecrubier Y, Sheehan KH, Janavs J, Weiller E, Keskiner A, et al. The validity of the Mini International Neuropsychiatric Interview (MINI) according to the SCID-P and its reliability. Eur Psychiatry 1997;12:232–41. https://doi.org/10.1016/S0924-9338(97)83297-X.

[27] Otsubo T, Tanaka K, Koda R, Shinoda J, Sano N, Tanaka S, et al. Reliability and validity of Japanese version of the Mini-International Neuropsychiatric Interview. Psychiatry Clin Neurosci 2005;59:517–26. https://doi.org/10.1111/j.1440-1819.2005.01408.x.

[28] Sheehan D v., Sheehan KH, Shytle RD, Janavs J, Bannon Y, Rogers JE, et al. Reliability and validity of the mini international neuropsychiatric interview for children and adolescents (MINI-KID). J Clin Psychiatry 2010;71:313–26. https://doi.org/10.4088/JCP.09m05305whi.

[29] Shittu RO, Issa BA, Olanrewaju GT, Mahmoud AO, Odeigah LO, Salami AK, et al. Prevalence and correlates of depressive disorders among people living with HIV/AIDS, in north central Nigeria. Journal of AIDS and Clinical Research 2013;4. https://doi.org/10.4172/2155-6113.1000251.

For peer review only

[30] Wang XB, Li XL, Zhang Q, Zhang J, Chen HY, Xu WY, et al. A survey of anxiety and depressive symptoms in pulmonary tuberculosis patients with and without tracheobronchial tuberculosis. Frontiers in Psychiatry 2018;9:1–10. https://doi.org/10.3389/fpsyt.2018.00308.

[31] Aamir S, Aisha. Co-morbid anxiety and depression among pulmonary tuberculosis patients. Journal of the College of Physicians and Surgeons Pakistan 2010;20:703–4. https://doi.org/10.2010/JCPSP.703704.

[32] Naidoo P, Mwaba K. Helplessness, depression, and social support among people being treated for tuberculosis in South Africa. Social Behavior and Personality 2010;38. https://doi.org/10.2224/sbp.2010.38.10.1323.

[33] Theron G, Peter J, Zijenah L, Chanda D, Mangu C, Clowes P, et al. Psychological distress and its relationship with non-adherence to TB treatment: A multicentre study. BMC Infect Dis 2015;15:253. https://doi.org/10.1186/s12879-015-0964-2.

[34] Xu M, Markström U, Lyu J, Xu L. Survey on tuberculosis patients in rural areas in China: tracing the role of stigma in psychological distress. Int J Environ Res Public Health 2017;14:1171. https://doi.org/10.3390/ijerph14101171.

[35] Chen X, Wu R, Xu J, Wang J, Gao M, Chen Y, et al. Prevalence and associated factors of psychological distress in tuberculosis patients in Northeast China: a cross-sectional study. BMC Infect Dis 2021;21:563. https://doi.org/10.1186/s12879-021-06284-4.

[36] Duko B, Bedaso A, Ayano G. The prevalence of depression among patients with tuberculosis: A systematic review and meta-analysis. Annals of General Psychiatry 2020;19:30. https://doi.org/10.1186/s12991-020-00281-8.

[37] Gooden TE, Gardner M, Wang J, Chandan JS, Beane A, Haniffa R, et al. The risk of mental illness in people living with HIV in the UK: a propensity score-matched cohort study. The Lancet HIV 2022;9. https://doi.org/10.1016/S2352-3018(21)00319-2.

For peer review only - http://bmjopen.bmj.com/site/about/guidelines.xhtml

[38] Too EK, Abubakar A, Nasambu C, Koot HM, Cuijpers P, Newton CRJC, et al. Prevalence and factors associated with common mental disorders in young people living with HIV in sub-Saharan Africa: a systematic review. J Int AIDS Soc 2021;24. https://doi.org/10.1002/jia2.25705.

[39] Nyongesa MK, Mwangi P, Kinuthia M, Hassan AS, Koot HM, Cuijpers P, et al. Prevalence, risk and protective indicators of common mental disorders among young people living with HIV compared to their uninfected peers from the Kenyan coast: a cross-sectional study. BMC Psychiatry 2021;21. https://doi.org/10.1186/s12888-021-03079-4.

[40] Myer L, Smit J, Roux L le, Parker S, Stein DJ, Seedat S. Common mental disorders among HIV-infected individuals in South Africa: Prevalence, predictors, and validation of brief psychiatric rating scales. AIDS Patient Care and STDs 2008;22. https://doi.org/10.1089/apc.2007.0102.

[41] Tesfaye SH, Bune GT. Generalized psychological distress among HIV-infected patients enrolled in antiretroviral treatment in Dilla University Hospital, Gedeo zone, Ethiopia. Global Health Action 2014;7. https://doi.org/10.3402/gha.v7.23882.

[42] Morrison SD, Banushi VH, Sarnquist C, Gashi VH, Osterberg L, Maldonado Y, et al. Levels of self-reported depression and anxiety among HIV-positive patients in Albania: A cross-sectional study. Croatian Medical Journal 2011;52. https://doi.org/10.3325/cmj.2011.52.622.

[43] Zewdu S. Common Mental Disorder among HIV Infected Individuals at Comprehensive HIV Care and Treatment Clinic of Debre Markos Referral Hospital, Ethiopia. Journal of AIDS & Clinical Research 2015;06. https://doi.org/10.4172/2155-6113.1000420.

For peer review only

[44] Evangeli M. Mental health and substance use in HIV-infected adolescents. Current Opinion in HIV and AIDS 2018;13. https://doi.org/10.1097/COH.0000000000000451.

[45] Woimo TT, Yimer WK, Bati T, Gesesew HA. The prevalence and factors associated for anti-tuberculosis treatment non-adherence among pulmonary tuberculosis patients in public health care facilities in South Ethiopia: a cross-sectional study. BMC Public Health 2017;17. https://doi.org/10.1186/s12889-017-4188-9.

[46] Ifebunandu NA, Ukwaja KN. Tuberculosis treatment default in a large tertiary care hospital in urban Nigeria: Prevalence, trend, timing and predictors. Journal of Infection and Public Health 2012;5. https://doi.org/10.1016/j.jiph.2012.06.002.

[47] Addis Z, Birhan W, Alemu A, Mulu A, Ayal G, Negash H. Treatment Outcome of Tuberculosis Patients in Azezo Health Center, North West Ethiopia. International Journal of Biomedical and Advance Research 2013;4. https://doi.org/10.7439/ijbar.v4i3.300.

[48] Tekle B, Mariam DH, Ali A. Defaulting from DOTS and its determinants in three districts of Arsi Zone in Ethiopia. International Journal of Tuberculosis and Lung Disease 2002;6.

[49] Lee LY, Tung HH, Chen SC, Fu CH. Perceived stigma and depression in initially diagnosed pulmonary tuberculosis patients. Journal of Clinical Nursing 2017;26. https://doi.org/10.1111/jocn.13837.

[50] Naidoo P, Peltzer K, Louw J, Matseke G, McHunu G, Tutshana B. Predictors of tuberculosis (TB) and antiretroviral (ARV) medication non-adherence in public primary care patients in South Africa: A cross sectional study. BMC Public Health 2013;13. https://doi.org/10.1186/1471-2458-13-396.

For peer review only

[51] Underner M, Perriot J, Peiffer G, Meurice J-C, Dautzenberg B. Smoking and adherence to anti-tuberculosis treatment. REVUE DES MALADIES RESPIRATOIRES 2016;33.

[52] Soboka M, Tesfaye M, Adorjan K, Krahl W, Tesfaye E, Yitayih Y, et al. Substance use disorders and adherence to antituberculosis medications in Southwest Ethiopia: A prospective cohort study. BMJ Open 2021;11. https://doi.org/10.1136/bmjopen-2020-043050.

[53] Muture BN, Keraka MN, Kimuu PK, Kabiru EW, Ombeka VO, Oguya F. Factors associated with default from treatment among tuberculosis patients in nairobi province, Kenya: A case control study. BMC Public Health 2011;11. https://doi.org/10.1186/1471-2458-11-696.

[54] Shargie EB, Lindtjørn B. Determinants of treatment adherence among smear-positive pulmonary tuberculosis patients in Southern Ethiopia. PLoS Medicine 2007;4. https://doi.org/10.1371/journal.pmed.0040037.

[55] Amuha MG, Kutyabami P, Kitutu FE, Odoi-Adome R, Kalyango JN. Non-adherence to anti-TB drugs among TB/HIV co-infected patients in Mbarara Hospital Uganda: prevalence and associated factors. Afr Health Sci 2009;9 Suppl 1.

[56] Husain MO, Dearman SP, Chaudhry IB, Rizvi N, Waheed W. Clinical Practice and Epidemiology The relationship between anxiety , depression and illness perception in tberculosis patients in Pakistan. Clinical Practice and Epidemiology in Mental Health 2008;5:4.

[57] Suhadev M, Thomas BE, Raja Sakthivel M, Murugesan P, Chandrasekaran V, Charles N, et al. Alcohol use disorders (AUD) among tuberculosis patients: A study from Chennai, South India. PLoS ONE 2011;6:e19485. https://doi.org/10.1371/journal.pone.0019485.

For peer review only

[58] Thapa P, Kamath R, Shetty BK, Monteiro A, Sekaran VC. Prevalence and Associated Factors of Alcoholism among Tuberculosis Patients in Udupi Taluk, Karnataka, India: A Cross Sectional Study. J Nepal Health Res Counc 2014;12:177–81.

[59] Peltzer K, Louw J, Mchunu G, Naidoo P, Matseke G, Tutshana B. Hazardous and harmful alcohol use and associated factors in tuberculosis public primary care patients in South Africa. International Journal of Environmental Research and Public Health 2012;9:3245–57. https://doi.org/10.3390/ijerph9093245.

[60] Necho M, Tsehay M, Seid M, Zenebe Y, Belete A, Gelaye H, et al. Prevalence and associated factors for alcohol use disorder among tuberculosis patients: a systematic review and meta-analysis study. Substance Abuse: Treatment, Prevention, and Policy 2021;16:2. https://doi.org/10.1186/s13011-020-00335-w.

[61] Veerakumar AM, Sahu SK, Sarkar S, Kattimani S, Govindarajan S. Alcohol use disorders among pulmonary tuberculosis patients under RNTCP in urban Pondicherry, India. Indian J Tuberc 2015;62:171–7. https://doi.org/10.1016/j.ijtb.2015.07.017.

[62] Taquet M, Geddes JR, Husain M, Luciano S, Harrison PJ. 6-month neurological and psychiatric outcomes in 236 379 survivors of COVID-19: a retrospective cohort study using electronic health records. The Lancet Psychiatry 2021;8:416–27. https://doi.org/10.1016/S2215-0366(21)00084-5.

[63] Lebina L, Dube M, Hlongwane K, Brahmbatt H, Lala SG, Reubenson G, et al. Trends in paediatric tuberculosis diagnoses in two south african hospitals early in the covid-19 pandemic. South African Medical Journal 2020;110:1149–50. https://doi.org/10.7196/SAMJ.2020.v110i12.15386.

For peer review only

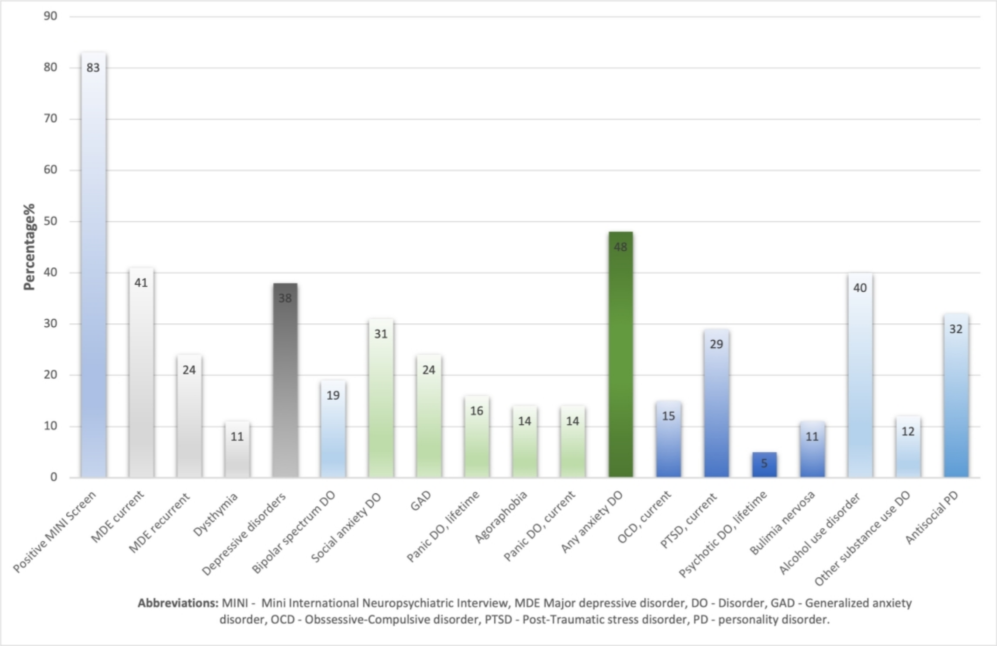

A proportion of patients with mental disorders among tuberculosis patients (N = 197)

239x155mm (300 x 300 DPI)

BMJ Open

The Co-Morbidity of Mental Ill-Health in Tuberculosis Patients under Treatment in a Rural Province of South Africa – A cross-sectional survey.

Journal:	*BMJ Open*
Manuscript ID	bmjopen-2021-058013.R3
Article Type:	Original research
Date Submitted by the Author:	27-Sep-2022
Complete List of Authors:	Thungana, Yanga; Walter Sisulu University, Department of Psychiatry and Behavioural Sciences<br>Wilkinson, Robert; Francis Crick Institute, Pathology; Imperial College London<br>Zingela, Zukiswa; Nelson Mandela University, Executive Dean's Office, Faculty of Health Sciences
**Primary Subject Heading**:	Mental health
Secondary Subject Heading:	Infectious diseases, Mental health, Public health
Keywords:	Tuberculosis < INFECTIOUS DISEASES, Substance misuse < PSYCHIATRY, Adult psychiatry < PSYCHIATRY, PUBLIC HEALTH

SCHOLARONE™
Manuscripts

BMJ

I, the Submitting Author has the right to grant and does grant on behalf of all authors of the Work (as defined in the below author licence), an exclusive licence and/or a non-exclusive licence for contributions from authors who are: i) UK Crown employees; ii) where BMJ has agreed a CC-BY licence shall apply, and/or iii) in accordance with the terms applicable for US Federal Government officers or employees acting as part of their official duties; on a worldwide, perpetual, irrevocable, royalty-free basis to BMJ Publishing Group Ltd ("BMJ") its licensees and where the relevant Journal is co-owned by BMJ to the co-owners of the Journal, to publish the Work in this journal and any other BMJ products and to exploit all rights, as set out in our licence.

The Submitting Author accepts and understands that any supply made under these terms is made by BMJ to the Submitting Author unless you are acting as an employee on behalf of your employer or a postgraduate student of an affiliated institution which is paying any applicable article publishing charge ("APC") for Open Access articles. Where the Submitting Author wishes to make the Work available on an Open Access basis (and intends to pay the relevant APC), the terms of reuse of such Open Access shall be governed by a Creative Commons licence – details of these licences and which Creative Commons licence will apply to this Work are set out in our licence referred to above.

Other than as permitted in any relevant BMJ Author's Self Archiving Policies, I confirm this Work has not been accepted for publication elsewhere, is not being considered for publication elsewhere and does not duplicate material already published. I confirm all authors consent to publication of this Work and authorise the granting of this licence.

For peer review only

The Co-Morbidity of Mental Ill-Health in Tuberculosis Patients under Treatment in a Rural Province of South Africa – A cross-sectional survey.

Yanga Thungana*[1], Robert John Wilkinson[2,3,4], Zukiswa Zingela[1]

1. Department of Psychiatry and Behavioural Sciences, Walter Sisulu University, Mthatha, South Africa
2. Wellcome Center for Infectious Diseases Research in Africa, Institute of Infect. Disease and Mol. Med and Dept. med. Univ. Cape Town, South Africa
3. Department of Infectious Disease, Imperial College, London, W12 ONN, UK
4. The Francis Crick Institute, London, NW1 IAT, UK

Correspondence address:

Yanga Thungana*

Department of psychiatry, Walter Sisulu University

Sisson street, Fort Gale, Mthatha, 5100

ythungana@wsu.ac.za

For peer review only

Abstract

Objectives: Tuberculosis (TB) remains prevalent despite the availability of effective anti-TB medications, and accumulating evidence suggests a high rate of mental disorders in people with TB. This is because TB and psychiatric disorders share several risk factors, such as poverty, homelessness, and substance use disorder. Moreover, psychiatric comorbidities in TB patients are associated with poor treatment outcomes. This study explored the psychiatric comorbidity and clinical correlates in individuals receiving TB treatment.

Design*:* A cross-sectional survey over ten months.

Setting: Two primary care clinics at King Sabata Dalindyebo District (KSD), Mthatha, Eastern Cape, South Africa.

Participants: Patients receiving TB treatment in the two clinics.

Interventions: The Mini-International Neuropsychiatric Interview was used to screen for psychiatric disorders.

Primary and Secondary Outcome Measures: Rates of mental disorders in TB patients over a ten-month period. Variation in rates by sex, employment status, and HIV comorbidity.

Results: In a sample of 197 participants, most patients were male (62%) and screened positive for a mental disorder (82%) with anxiety (48%), depression (38%), and substance use disorders (43%) being the most common psychiatric conditions. On average, individuals had 4 (SD 2) mental disorders. Females had higher rates of depression ($p = 0.005$) and nonadherence to TB treatment ($p = 0.003$), and alcohol use disorder was more common in males ($p < 0.001$) and in those nonadherent to TB treatment. Additionally, low education levels and unemployment were associated with depressive and anxiety disorders ($p < 0.05$).

Conclusions: Mental disorders are common in patients with tuberculosis, and mental health services need to be integrated into the management of patients with TB. Factors linked to mental disorders in this cohort, such as low education, gender, and unemployment, may be

For peer review only

useful for compiling a risk profile to help identify those with TB who may require more intensive support for their mental health.

Strengths and limitations of this study

- Using a structured clinical diagnostic interview improved consistency in the individual assessments.
- The MINI covers a broad range of mental disorders, and the most common psychiatric disorders are included in the scale.
- As a cross-sectional study, causal inferences could not be drawn between variables
- The inclusion of only rural participants and a small sample could limit the generalizability of study findings

For peer review only

Despite the availability of effective anti-tuberculosis drugs, tuberculosis (TB) remains one of the top ten causes of death worldwide, and it is among the leading causes of death from a single infectious agent. [1] South Africa is among the top eight countries that account for two-thirds of the global TB infections. [1] Additionally, in people infected with TB, there are high rates of mental disorders such as depression and anxiety disorders. [2–5] Depression is one of the most common comorbid psychiatric disorders in people with TB and is estimated to be 3-times higher than in patients without TB. [6–8] Conversely, people with psychiatric disorders may be at an increased risk of developing TB as well. [9–11]

There are several postulated reasons for the increased prevalence of mental disorders in people with TB, including shared risk factors such as homelessness, HIV/AIDS, substance use, stigma, malnutrition, and poor socioeconomic status. [12–15] Psychiatric comorbidity in TB is associated with poor treatment outcomes, disability, treatment failure, and poor quality of life due to poor health-seeking behaviours and challenges with adherence to medication in this cohort of patients. [16–18] Substance use, especially alcohol use, is prevalent in patients with tuberculosis and is thought to increase TB incidence, TB mortality, delayed presentation to health facilities, and poor adherence to TB treatment. [19–21] Furthermore, there is evidence that treating comorbid psychiatric disorders can improve TB outcomes. [22]

Although the relationship between TB and mental disorders is described, there is often a lack of integration of mental health services in the management of patients with TB. [23] Additionally, in South Africa, limited studies have explored in detail the prevalence of psychiatric disorders in people with TB. The value of this study is in the use of a different psychiatry questionnaire (Mini-International Neuropsychiatric Interview) that is more extensive and evaluates many more different psychiatric disorders compared to previous

For peer review only

studies evaluating mental disorders in TB patients. Also, unique to our study is that the MINI is observer administered compared to the commonly used self-report questionnaires. Therefore, we sought to investigate the point prevalence of comorbid mental disorders and its clinical correlates in patients receiving TB treatment in two district clinics in a rural area in the Eastern Cape, South Africa.

METHODS

Sample and procedure

A cross-sectional study was conducted among TB patients attending two primary care clinics (Mthatha Gateway and Stanford Terrace) at King Sabata Dalindyebo District (KSD), Mthatha, Eastern Cape, South Africa. Both clinics offer general medical care, including TB screening and the management of patients with TB. The study was conducted over a ten-month period, from September 2020 to June 2021. Participants included in the study were all patients currently diagnosed and receiving TB treatment who attended the two clinics during the study period. In these clinics, each morning, a nurse holds a short meeting with patients attending the clinic on the day to triage and guide them on accessing services. It is in these morning meetings that the nurse would identify all patients coming to collect tuberculosis treatment and would inform the research assistant (RA), who then approached each patient individually. Due to the coronavirus (Covid-19) pandemic and applicable South African lockdown restrictions at the time, the total number of patients attending clinics was significantly reduced.

For peer review only

The RA would explain the study to each patient individually in their language, obtain written informed consent, and assure them that their personally identifying information would be kept confidential at all times. Excluded from the study were participants under 18 years of age and those who could not give informed consent. A total of 197 participants were approached by the RA; all provided written consent and thus were included in the study. For our study, a mental disorder, also referred to as a psychiatric disorder, is a condition with a clinically significant disturbance of an individual's emotions, cognition, or behaviour associated with personal distress or impairment in adaptive function. And would include all the conditions recognized in the DSM-5. Psychiatric comorbidity or comorbid psychiatric disorder in our study refers to diagnosing a mental disorder in a patient with current tuberculosis.

Data collection

The RA had prior experience in mental health research and was trained on using the MINI over ten working days by a psychiatrist. The training was held for 90 minutes each day regarding how to use the different modules of the MINI and an explanation of the various psychiatric terms used in the MINI. The last two sessions were role-playing interviews to assess the RA's level of understanding of the MINI. Lastly, the RA received supervision throughout the period of data collection from the lead author, who is a qualified specialist psychiatrist and a neuropsychiatrist fellow at the time of the study.

The English version of the MINI was administered in English to all study participants. The RA was instructed to read the questions slowly, repeat them as necessary, and encourage the patients to seek clarity on any question that is not entirely clear to them. The RA was

For peer review only

instructed to schedule at least an hour to accommodate those slow in answering the numerous questions of the MINI. Lastly, when a patient did not understand a question, the RA was allowed to explain it in the patient's primary language.

Measures

A data capture sheet was used to collect sociodemographic details, past psychiatric history, medical history including HIV status, and adherence to TB treatment. Data on whether a participant had defaulted on their current TB treatment were also sought from the patient, although their responses were not verified through other means. The patients were asked whether they had stopped taking the TB treatment for at least 7 consecutive days.

The Mini-International Neuropsychiatric Interview (MINI) was developed as a brief, structured diagnostic interview used as a measure to diagnose several psychiatric disorders. [24] It has demonstrated good psychometric properties as a tool to screen for several psychiatric disorders. [24–26] For instance, the specificity of the MINI is at least good to excellent (0.72 – 1.0) for the diverse conditions screened is used to evaluate. [25,27,28] Although the MINI contains over 100 questions and screens for 17 mental disorders, it can be administered over a short period – approximately 20 – 30 minutes. The MINI is organised into diagnostic modules. For nearly all the modules, two to four screening questions are used to exclude a diagnosis if answered negatively. Conversely, positive responses to the screening questions lead to further inquiry about other diagnostic criteria. The MINI modules include a major depressive disorder (current and recurrent), dysthymia, manic & hypomanic episodes (current and past), panic disorder (current and lifetime), agoraphobia, social phobia, obsessive-compulsive disorder, post-traumatic stress disorder, alcohol abuse & dependence,

For peer review only

non-alcohol substance use & dependence, psychotic disorders (current and lifetime), anorexia nervosa, bulimia nervosa, generalized anxiety disorder, and antisocial personality disorder. We used all the MINI modules in our study.

Patient and public involvement

There was no involvement of patients in the design, recruitment, and conduct of the study. The study results will not be distributed to the individual participants, but the published paper will be available in the participating clinics.

Data analytical plan

Data were analysed using STATA/SE "(version 16.1 for Mac)". Descriptive statistics such as frequencies and proportions for categorical variables, median and interquartile range (IQR), or mean and standard deviation for continuous variables depending on the data skewness were utilised to characterise the sample. Inferential statistics were applied to evaluate the association between different variables. Firstly, to assess associations between categorical variables, we performed either the Pearson's chi-square test or Fischer's exact test, depending on the number of scored items under each variable. Secondly, for the continuous variables, we first assessed for normality using the Skewness and Kurtosis test (*sktest*) with a $p > 0.05$ indicating normal distribution. Therefore, due to the skewness of our data, we used either the Wilcoxon rank-sum test or the Kruskal-Wallis test to evaluate associations between numerical and categorical variables. The significance level was set at $P < 0.05$ for all the bi-variate hypothesis testing.

For peer review only

RESULTS

Most of the 197 participants were men (61.9%, n = 122), and the median age was 34 years (IQR 28-42) of age (**Table 1**). Most of the participants were single (70.6%, n = 139), had at least secondary education (56.9%, n= 112), and were unemployed (57.4%, n = 113). We did not find any statistically significant associations between age and the different psychiatric disorders. For instance, the adult group (44%, n = 66) had the highest proportion of participants with an alcohol use disorder compared to other age groups, but it was not statistically significant (p = 0.055). Unemployed participants were more likely to have an anxiety disorder (p = 0.03), depressive disorder (p < 0.001), and obsessive-compulsive disorder (OCD) (p = 0.006). Moreover, individuals earning less than R2500.00 a month had a higher proportion of depressive disorders (74.7%, p < 0.001), social phobia (75.4%, p = 0.03), and agoraphobia (85.7%, p = 0.01) than those earning more than R2500.00. Lower levels of education were associated with recurrent depressive disorder (P = 0.001), lifetime panic disorder (p = 0.02), ongoing generalized anxiety disorder (p = 0.02), and current OCD (p = 0.03). But social anxiety disorder was more common in those with at least a high school education (p = 0.02).

Table 1. Sociodemographic details of the participants (N=197)

	Number	Percentage
Sex		
Male	122	61.9%
Female	75	38.1%
Age		
Adolescents (<19 years)	4	2%
Adults (19 – 44)	149	76%
Middle age (45 – 64)	33	16.8%

For peer review only

Aged+ (65+)	10	5.1%
**Relationship status**		
Single	139	70.6%
Married	44	22.3%
Divorced/separated	14	7.1%
**Level of education**		
Primary school	33	16.8%
High school	112	56.9%
Tertiary education	52	26.4%
**Employment status**		
Employed	84	42.6%
Unemployed	113	57.4%
Receiving state grant/pension	26	13.2%
**Monthly income[a]**		
Less than R2 500	127	64.5%
More than R2 500	70	35.5%
**HIV status**		
Negative	62	31.5%
Positive	128	64.97%
Unknown	7	3.6%
**Taking ART**		
Yes	99	77.3%
No	29	22.6%
**Disease classification**		
Pulmonary	153	77.7%
Extrapulmonary	44	22.3%
**Phase of TB treatment**		
Active	102	51.8%
Continuation	95	48.2%

Abbreviations: ART - Antiretroviral therapy; TB - Tuberculosis. [a] - SA Poverty line is R1 220

Medical history

For peer review only

The majority of the participants were HIV positive (65%, n = 128) (95% CI 58.0 - 71.4) with significantly higher proportion of males (78.4%, n = 58) having HIV than females (60.7, n = 71) participants (p = 0.01). Most participants with an HIV diagnosis were receiving antiretroviral therapy (77.3%, n = 99) (**Table 1**). Most HIV-positive (96.6%, n = 28) participants not on ART were still in the intensive phase of TB treatment. Moreover, most of the participants in the sample (77.7%, n = 153) had pulmonary TB, and more than half (51.8%, n = 102) were in the intensive phase of TB treatment. Only a minority of patients (9.1%, n = 18) reported non-adherence to their TB treatment. Most participants reporting non-adherence to TB treatment were HIV positive (77.8%, p = 0.4), still in the intensive phase (55.6%, p = 0.8) of TB treatment, unemployed (55.6%, p = 0.8), and assessed to have a mental disorder (88.9%, p = 0.4) but these did not reach statistical significance. However, being female (p = 0.003) and having an alcohol use disorder (p = 0.001) were significantly associated with non-adherence to TB treatment.

Mental disorders

Most of the participants (82.7%, n = 163) screened positive for at least one psychiatric disorder, excluding substance use disorders (***Fig 1***), and the proportion of males (82.1%, n = 101) with a positive screen was similar to that of female (84%, n = 63) participants. Furthermore, the HIV status of the participants was not associated with screening outcomes for psychiatric disorders, with those HIV negative almost screening equally positive for a psychiatric disorder as those who were HIV positive (80.7% HIV-negative vs. 85.2% HIV-positive; p = 0.4). There was a comparable proportion of individuals assessed to have a psychiatric diagnosis in those in the intensive phase (82.5%, n = 85) and a continuation phase (83.2%, n = 79) of TB treatment.

For peer review only

The most common psychiatric disorders were anxiety disorders (48.2%, n = 95), current major depressive episodes (40.6%, n = 80), unipolar depression (37.6%, n = 74), post-traumatic stress disorder (28.9%, n = 57), antisocial personality (23.9%, n = 47), and bipolar spectrum conditions (19.3%, n = 38). Approximately a quarter (25.7%, n = 19) of the patients with unipolar depression currently had suicidal ideation. The patients with a positive diagnosis of a mental disorder had an average of 3.5 (SD 2.3) disorders excluding substance use disorders. Also, patients tended to have more than one anxiety disorder with an average of 2 (SD 1) disorders per individual.

More participants (47%) in the intensive phase of TB treatment had a current depressive episode compared to those in the continuation phase (34%); however, this was not statistically significant ($p = 0.056$). Noteworthily, female participants were more likely to have a depressive disorder than male participants ($p = 0.005$). In contrast, males were more likely to screen positive for an antisocial personality disorder ($p < 0.001$).

Substance use disorders

A considerable number of the participants had a substance use disorder (43.2%, n = 85), with more participants having an alcohol use disorder (40.1%, n = 79) than those having a substance use disorder other than alcohol (12.2%, n = 24). However, most of the 24 participants (79.2%, n = 19) with other substance use disorders also had alcohol use disorder. Nevertheless, both alcohol use disorders ($p = 0.001$) and other substance use disorders ($p = 0.001$) were more common among male participants. In this sample, there was no significant association between HIV status and substance use disorders.

For peer review only

DISCUSSION

In this study, we examined the psychiatric comorbidity in outpatients receiving TB treatment. We found a high rate of mental disorders in patients with TB; depression, anxiety, and substance use disorders were the most common mental disorders. Additionally, those with mental disorders approximately had 4 (SD 2) lifetime psychiatric disorders, excluding substance use disorders. Also, alcohol use disorder was prevalent in our study participants and was significantly associated with the male sex and poor adherence to TB treatment. Female sex was associated with depressive disorders and nonadherence to treatment. Lastly, low levels of education and unemployment were associated with a higher rate of depressive, anxiety, and obsessive-compulsive disorders.

The prevalence of psychiatric disorders in patients with tuberculosis varies greatly among different studies from less than 20% to about 80% with a trend of higher rates in lower-middle-income countries (LMIC) [21,29–31]. In South Africa (SA), the prevalence of psychiatric comorbidity in TB patients is similar to other LMIC. For instance, the prevalence of depression is documented to be 64.3%, and PTSD at 29.6%.[6,32] But higher rates of psychological distress (81%) have been reported in SA.[6,7] In our study, we found an overall higher rate of mental disorders (approx. 83%), but the rates of PTSD and depression were similar and lower to previous studies, respectively.[6,32] The major variation in prevalence in our study could be due to several factors such as the assessment instrument used, a high HIV comorbidity, the sociodemographic factors, or that it is a true reflection of the high psychiatric comorbidity in this population. For instance, HIV comorbidity in patients with TB is associated with higher mental morbidity.[33] Also, South Africa is a middle-

For peer review only - http://bmjopen.bmj.com/site/about/guidelines.xhtml

income country with a very high TB burden; the personal socioeconomic consequences of TB infection might be amplified, thus leading to more pronounced mental disorders. [1,33] A case in point documented is that in TB patients, unemployment, poverty, and lower educational background are associated with higher psychiatric comorbidity, as was the case in our study. [6,34,35]

Furthermore, the MINI covers a broader number of conditions than scales used in other studies, and in patients with TB, mental disorders have been found to vary significantly depending on the psychiatric screening scale used. [36] Compared to previous studies that tended to assess broader categories of mental disturbance such as anxiety or psychological distress, we were able to screen for several individual psychiatric diagnoses. Thus, in addition to describing the presence of mental illness, we were able to assess the number of disorders experienced by each individual and we found a tendency for participants to have more than one psychiatric disorder with an average of approx. 4 (SD 2) disorders per individual, excluding substance use disorders.

In this study, we did not find a statistically significant relationship between HIV-positive individuals and an increased rate of mental disorders. But the finding of increased psychiatric disorders in PLWH has been described. [37–39] However, the rate of mental illness in PLWH varies significantly from as low as below 10% to as high as over 80%. [29,40–43] The wide range in the prevalence could be due to the different study designs, study settings, and measurement instruments used in different studies. Moreover, not all studies have found an increased rate of mental illness in PLWH. [44]

For peer review only

We found a slightly higher adherence rate to TB treatment (91%) in the current study. Previous studies have documented adherence rates between 70% and 89%. [45–48] The higher rates of treatment adherence in our study could be due to our methodology, as we solely relied on the patient's report and did investigate it further. Non-adherence to treatment in patients with TB can be due to several factors, including HIV seropositivity, substance use, distance to health facilities, male gender, running out of medication, medication factors, the continuation phase of chemotherapy, and stigma. [45–47] Additionally, psychiatric comorbidity has been described as another factor with a negative impact on compliance with TB treatment.[5,33,49,50] Also, substance use is associated with poor adherence to TB treatment,[50–52] as was the case in our study. Contrary to findings in the current study, previous studies have found better adherence to tuberculosis treatment among female participants.[47,53] Furthermore, the proportion of participants with psychiatric disorders was comparable between those in the intensive phase and continuation phase of chemotherapy. But the continuation phase of TB treatment is previously associated with a higher rate of mental illness.[48,54,55]

Our finding that anxiety and depressive disorders are the most common mental disorders in patients with TB is comparable to previous studies. [21,36,56] Moreover, as in our study, women with TB were more likely to suffer from depressive disorders than male patients.[33,36] It has been reported that psychiatric disorders are more common in the earlier phase of TB infection and rates decline as patients recover from TB infection.[21] Our study showed a similar trend with more patients in the intensive phase of treatment having a current major depressive episode than those in the continuation phase; however, the difference did not reach statistical significance.

For peer review only

Alcohol use disorder was common in our study sample, corroborating a finding in previous literature highlighting high rates of alcohol use in patients with TB.[57–60] In those already infected with TB, alcohol abuse is associated with a lack of cooperation, poor adherence, higher levels of depression, and mortality.[19,20] Similar to previous studies, alcohol use disorders were more common among the male participants in our study.[59,61] However, the prevalence of alcohol use disorder (40%) in our study was higher than previously reported and could be because most participants were males, single, unemployed, and had lower education levels.[57,60]

Strengths and limitations of this study

The use of the MINI, a well-established structured diagnostic interview scale that covers a broader number of mental disorders, adds to the study's strength. Furthermore, all patients with TB attending the two clinics were admitted to the study with no exclusion criteria. The study was conducted during the COVID-19 pandemic, which possibly affected clinic attendance and TB screening and could have contributed to the high prevalence of mental disorders.[62,63] Along with this, the small study sample could restrain the generalizability of the study results to other settings. Additionally, we did not conduct individual clinical assessments to confirm the diagnosis of the MINI, which could possibly increase the positive psychiatric diagnosis. Lastly, the MINI was administered in English to participants to whom English is not their primary language, which might have affected the accuracy of the patient's responses.

CONCLUSION

For peer review only

Our study showed an increased frequency and number of mental disorders among individuals receiving TB treatment. Moreover, as in previous studies, we found substance use disorders prevalent in TB patients. Thus, the study outcomes further highlight the need to integrate mental health services in managing patients with TB at the primary care level, including screening all new TB patients. There is a need for studies to evaluate whether brief interventions for substance use at the primary care level could improve adherence and recovery from tuberculosis.

ETHICS STATEMENTS

Patient consent for publication

Not required

Ethics approval

Ethical approval for this study was obtained from the Walter Sisulu University Human Research Committee (protocol number 119/2019) and the South African Department of Health (EC_202006_009). Each participant, before taking part in the study, gave a signed informed consent in their primary language of communication.

ACKNOWLEDGEMENTS

We would like to express our gratitude to our research assistant, Simthembile Lindani, who worked tirelessly and completed data collection despite the challenges of the COVID-19 pandemic. We would like to thank Editage (www.editage.com) for English language editing.

COMPETING INTERESTS

All authors have completed the ICMJE uniform disclosure form and declare no conflict of interest.

FUNDING

For peer review only

This research was Supported by the National Institutes of Health grant number (NIH U19AI115940). RJW is supported by the Francis Crick Institue which receives funding from Wellcome (FC0010218), Cancer Research UK (FC0010218), and the Medical Research Council (FC0010218). For the purpose of open access, the authors have applied a CC-BY public copyright to any author-accepted manuscript arising from this submission.

DATA SHARING STATEMENT

The data (deidentified participants dataset) can be available upon reasonable request from the corresponding author.

AUTHORS' CONTRIBUTIONS

YT, RJW, and ZZ were involved in the conception and design of the study. YT analysed the data, and YT wrote the manuscript. YT, RJW, and ZZ revised the manuscript. All authors provided their final approval for manuscript submission and agree to be accountable for all aspects of the work. YT, as the corresponding author, confirms that all authors meet the authorship criteria and that no others have been omitted.

Table 1: The sociodemographic details of the survey sample

Figure 1: A proportion of patients with mental disorders among tuberculosis patients (N = 197)

For peer review only

REFERENCES

[1] Global tuberculosis report. Geneva: World Health Organization; 2020. International Journal of Infectious Diseases 2021. https://doi.org/10.1016/j.ijid.2021.02.107.

[2] Mason PH, Sweetland AC, Fox GJ, Halovic S, Nguyen TA, Marks GB. Tuberculosis and mental health in the Asia-Pacific. Australasian Psychiatry 2016;24:553–5. https://doi.org/10.1177/1039856216649770.

[3] Koyanagi A, Vancampfort D, Carvalho AF, DeVylder JE, Haro JM, Pizzol D, et al. Depression comorbid with tuberculosis and its impact on health status: cross-sectional analysis of community-based data from 48 low- and middle-income countries. BMC Med 2017;15:209. https://doi.org/10.1186/s12916-017-0975-5.

[4] Doherty AM, Kelly J, McDonald C, O'Dywer AM, Keane J, Cooney J. A review of the interplay between tuberculosis and mental health. Gen Hosp Psychiatry 2013;35:398–406. https://doi.org/10.1016/j.genhosppsych.2013.03.018.

[5] Ruiz-Grosso P, Cachay R, de La Flor A, Schwalb A, Ugarte-Gil C. Association between tuberculosis and depression on negative outcomes of tuberculosis treatment: a systematic review and meta-analysis. PLoS ONE 2020;15:e0227472. https://doi.org/10.1371/journal.pone.0227472.

[6] Peltzer K, Naidoo P, Matseke G, Louw J, McHunu G, Tutshana B. Prevalence of post-traumatic stress symptoms and associated factors in tuberculosis (TB), TB retreatment and/or TB-HIV co-infected primary public health-care patients in three districts in South Africa. Psychol Health Med 2013;18:387–97. https://doi.org/10.1080/13548506.2012.726364.

[7] Peltzer K, Naidoo P, Matseke G, Louw J, Mchunu G, Tutshana B. Prevalence of psychological distress and associated factors in tuberculosis patients in public primary

For peer review only

care clinics in South Africa. BMC Psychiatry 2012;12:89. https://doi.org/10.1186/1471-244X-12-89.

[8] de Araújo GS, Pereira SM, dos Santos DN, Marinho JM, Rodrigues LC, Barreto ML. Common mental disorders associated with tuberculosis: a matched case-control study. PLoS ONE 2014;9:e99551. https://doi.org/10.1371/journal.pone.0099551.

[9] Mcquistlon L, Susser E. Tuberculosis infection among people with severe mental illness. Psychiatr Serv 1997;48:833–5. https://doi.org/10.1176/ps.48.6.833.

[10] Oh KH, Choi H, Kim EJ, Kim HJ, Cho SI. Depression and risk of tuberculosis: a nationwide populationbased cohort study. Inte J Tuberc Lung Dis 2017;21:804–9. https://doi.org/10.5588/ijtld.17.0038.

[11] Cheng KC, Liao KF, Lin CL, Lai SW. Increased risk of pulmonary tuberculosis in patients with depression: a cohort study in Taiwan. Frontiers in Psychiatry 2017;8:235. https://doi.org/10.3389/fpsyt.2017.00235.

[12] Pachi A, Bratis D, Moussas G, Tselebis A. Psychiatric morbidity and other factors affecting treatment adherence in pulmonary tuberculosis patients. Tuberculosis Research and Treatment 2013;2013:489865. https://doi.org/10.1155/2013/489865.

[13] Duarte EC, Bierrenbach AL, da Silva JB, Tauil PL, de Fátima Duarte E. Factors associated with deaths among pulmonary tuberculosis patients: a case control study with secondary data. Journal of Epidemiology and Community Health 2009;63:233–8. https://doi.org/10.1136/jech.2008.078972.

[14] Martens WHJ. A review of physical and mental health in homeless persons. Public Health Rev 2001;29:13–33.

[15] Oeltmann JE, Kammerer JS, Pevzner ES, Moonan PK. Tuberculosis and Substance Abuse in the United States, 1997-2006. Arch Intern Med 2009;169:189–97. https://doi.org/10.1001/archinternmed.2008.535.

For peer review only

[16] Simon GE, von Korff M, Lin E. Clinical and functional outcomes of depression treatment in patients with and without chronic medical illness. Psychol Med 2005;35:271–9. https://doi.org/10.1017/S0033291704003071.

[17] Ambaw F, Mayston R, Hanlon C, Medhin G, Alem A. Untreated depression and tuberculosis treatment outcomes, quality of life and disability, Ethiopia. Bull World Health Organ 2018;96:243–55. https://doi.org/10.2471/BLT.17.192658.

[18] Sweetland AC, Kritski A, Oquendo MA, Sublette ME, Norcini Pala A, Silva LRB, et al. Addressing the tuberculosis–depression syndemic to end the tuberculosis epidemic. Intern J Tuberc Lung Dis 2017;2:852–61. https://doi.org/10.5588/ijtld.16.0584.

[19] Lackey B, Seas C, van der Stuyft P, Otero L. Patient characteristics associated with tuberculosis treatment default: a cohort study in a high-incidence area of Lima, Peru. PLoS ONE 2015;10:e0128541. https://doi.org/10.1371/journal.pone.0128541.

[20] Imtiaz S, Shield KD, Roerecke M, Samokhvalov A v., Lönnroth K, Rehm J. Alcohol consumption as a risk factor for tuberculosis: meta-analyses and burden of disease. Eur Respir J 2017;50:1700216. https://doi.org/10.1183/13993003.00216-2017.

[21] Shyamala KK, Sharadha Naveen R, Khatri B. Depression: A neglected comorbidity in patients with tuberculosis. Journal of Association of Physicians of India 2018;66:18–21.

[22] Acha J, Sweetland A, Guerra D, Chalco K, Castillo H, Palacios E. Psychosocial support groups for patients with multidrug-resistant tuberculosis: five years of experience. Global Public Health 2007;2:404–17. https://doi.org/10.1080/17441690701191610.

[23] Walker IF, Baral SC, Wei X, Huque R, Khan A, Walley J, et al. Multidrug-resistant tuberculosis treatment programmes insufficiently consider comorbid mental disorders. Intern J Tuberc Lung Dis 2017;21:603–9. https://doi.org/10.5588/ijtld.17.0135.

For peer review only

[24] Sheehan D v., Lecrubier Y, Sheehan KH, Amorim P, Janavs J, Weiller E, et al. The Mini-International Neuropsychiatric Interview (M.I.N.I.): The development and validation of a structured diagnostic psychiatric interview for DSM-IV and ICD-10. J Clin Psychiatry, vol. 59, 1998, p. 22–33.

[25] Lecrubier Y, Sheehan D v., Weiller E, Amorim P, Bonora I, Sheehan KH, et al. The Mini International Neuropsychiatric Interview (MINI): a short diagnostic structured interview: Reliability and validity according to the CIDI. Eur Psychiatry 1997;12:224–31. https://doi.org/10.1016/S0924-9338(97)83296-8.

[26] Sheehan D v., Lecrubier Y, Sheehan KH, Janavs J, Weiller E, Keskiner A, et al. The validity of the Mini International Neuropsychiatric Interview (MINI) according to the SCID-P and its reliability. Eur Psychiatry 1997;12:232–41. https://doi.org/10.1016/S0924-9338(97)83297-X.

[27] Otsubo T, Tanaka K, Koda R, Shinoda J, Sano N, Tanaka S, et al. Reliability and validity of Japanese version of the Mini-International Neuropsychiatric Interview. Psychiatry Clin Neurosci 2005;59:517–26. https://doi.org/10.1111/j.1440-1819.2005.01408.x.

[28] Sheehan D v., Sheehan KH, Shytle RD, Janavs J, Bannon Y, Rogers JE, et al. Reliability and validity of the mini international neuropsychiatric interview for children and adolescents (MINI-KID). J Clin Psychiatry 2010;71:313–26. https://doi.org/10.4088/JCP.09m05305whi.

[29] Shittu RO, Issa BA, Olanrewaju GT, Mahmoud AO, Odeigah LO, Salami AK, et al. Prevalence and correlates of depressive disorders among people living with HIV/AIDS, in north central Nigeria. Journal of AIDS and Clinical Research 2013;4. https://doi.org/10.4172/2155-6113.1000251.

For peer review only

[30] Wang XB, Li XL, Zhang Q, Zhang J, Chen HY, Xu WY, et al. A survey of anxiety and depressive symptoms in pulmonary tuberculosis patients with and without tracheobronchial tuberculosis. Frontiers in Psychiatry 2018;9:1–10. https://doi.org/10.3389/fpsyt.2018.00308.

[31] Aamir S, Aisha. Co-morbid anxiety and depression among pulmonary tuberculosis patients. Journal of the College of Physicians and Surgeons Pakistan 2010;20:703–4. https://doi.org/10.2010/JCPSP.703704.

[32] Naidoo P, Mwaba K. Helplessness, depression, and social support among people being treated for tuberculosis in South Africa. Social Behavior and Personality 2010;38. https://doi.org/10.2224/sbp.2010.38.10.1323.

[33] Theron G, Peter J, Zijenah L, Chanda D, Mangu C, Clowes P, et al. Psychological distress and its relationship with non-adherence to TB treatment: A multicentre study. BMC Infect Dis 2015;15:253. https://doi.org/10.1186/s12879-015-0964-2.

[34] Xu M, Markström U, Lyu J, Xu L. Survey on tuberculosis patients in rural areas in China: tracing the role of stigma in psychological distress. Int J Environ Res Public Health 2017;14:1171. https://doi.org/10.3390/ijerph14101171.

[35] Chen X, Wu R, Xu J, Wang J, Gao M, Chen Y, et al. Prevalence and associated factors of psychological distress in tuberculosis patients in Northeast China: a cross-sectional study. BMC Infect Dis 2021;21:563. https://doi.org/10.1186/s12879-021-06284-4.

[36] Duko B, Bedaso A, Ayano G. The prevalence of depression among patients with tuberculosis: A systematic review and meta-analysis. Annals of General Psychiatry 2020;19:30. https://doi.org/10.1186/s12991-020-00281-8.

[37] Gooden TE, Gardner M, Wang J, Chandan JS, Beane A, Haniffa R, et al. The risk of mental illness in people living with HIV in the UK: a propensity score-matched cohort study. The Lancet HIV 2022;9. https://doi.org/10.1016/S2352-3018(21)00319-2.

For peer review only

[38] Too EK, Abubakar A, Nasambu C, Koot HM, Cuijpers P, Newton CRJC, et al. Prevalence and factors associated with common mental disorders in young people living with HIV in sub-Saharan Africa: a systematic review. J Int AIDS Soc 2021;24. https://doi.org/10.1002/jia2.25705.

[39] Nyongesa MK, Mwangi P, Kinuthia M, Hassan AS, Koot HM, Cuijpers P, et al. Prevalence, risk and protective indicators of common mental disorders among young people living with HIV compared to their uninfected peers from the Kenyan coast: a cross-sectional study. BMC Psychiatry 2021;21. https://doi.org/10.1186/s12888-021-03079-4.

[40] Myer L, Smit J, Roux L le, Parker S, Stein DJ, Seedat S. Common mental disorders among HIV-infected individuals in South Africa: Prevalence, predictors, and validation of brief psychiatric rating scales. AIDS Patient Care and STDs 2008;22. https://doi.org/10.1089/apc.2007.0102.

[41] Tesfaye SH, Bune GT. Generalized psychological distress among HIV-infected patients enrolled in antiretroviral treatment in Dilla University Hospital, Gedeo zone, Ethiopia. Global Health Action 2014;7. https://doi.org/10.3402/gha.v7.23882.

[42] Morrison SD, Banushi VH, Sarnquist C, Gashi VH, Osterberg L, Maldonado Y, et al. Levels of self-reported depression and anxiety among HIV-positive patients in Albania: A cross-sectional study. Croatian Medical Journal 2011;52. https://doi.org/10.3325/cmj.2011.52.622.

[43] Zewdu S. Common Mental Disorder among HIV Infected Individuals at Comprehensive HIV Care and Treatment Clinic of Debre Markos Referral Hospital, Ethiopia. Journal of AIDS & Clinical Research 2015;06. https://doi.org/10.4172/2155-6113.1000420.

For peer review only

[44] Evangeli M. Mental health and substance use in HIV-infected adolescents. Current Opinion in HIV and AIDS 2018;13. https://doi.org/10.1097/COH.0000000000000451.

[45] Woimo TT, Yimer WK, Bati T, Gesesew HA. The prevalence and factors associated for anti-tuberculosis treatment non-adherence among pulmonary tuberculosis patients in public health care facilities in South Ethiopia: a cross-sectional study. BMC Public Health 2017;17. https://doi.org/10.1186/s12889-017-4188-9.

[46] Ifebunandu NA, Ukwaja KN. Tuberculosis treatment default in a large tertiary care hospital in urban Nigeria: Prevalence, trend, timing and predictors. Journal of Infection and Public Health 2012;5. https://doi.org/10.1016/j.jiph.2012.06.002.

[47] Addis Z, Birhan W, Alemu A, Mulu A, Ayal G, Negash H. Treatment Outcome of Tuberculosis Patients in Azezo Health Center, North West Ethiopia. International Journal of Biomedical and Advance Research 2013;4. https://doi.org/10.7439/ijbar.v4i3.300.

[48] Tekle B, Mariam DH, Ali A. Defaulting from DOTS and its determinants in three districts of Arsi Zone in Ethiopia. International Journal of Tuberculosis and Lung Disease 2002;6.

[49] Lee LY, Tung HH, Chen SC, Fu CH. Perceived stigma and depression in initially diagnosed pulmonary tuberculosis patients. Journal of Clinical Nursing 2017;26. https://doi.org/10.1111/jocn.13837.

[50] Naidoo P, Peltzer K, Louw J, Matseke G, McHunu G, Tutshana B. Predictors of tuberculosis (TB) and antiretroviral (ARV) medication non-adherence in public primary care patients in South Africa: A cross sectional study. BMC Public Health 2013;13. https://doi.org/10.1186/1471-2458-13-396.

For peer review only

[51] Underner M, Perriot J, Peiffer G, Meurice J-C, Dautzenberg B. Smoking and adherence to anti-tuberculosis treatment. REVUE DES MALADIES RESPIRATOIRES 2016;33.

[52] Soboka M, Tesfaye M, Adorjan K, Krahl W, Tesfaye E, Yitayih Y, et al. Substance use disorders and adherence to antituberculosis medications in Southwest Ethiopia: A prospective cohort study. BMJ Open 2021;11. https://doi.org/10.1136/bmjopen-2020-043050.

[53] Muture BN, Keraka MN, Kimuu PK, Kabiru EW, Ombeka VO, Oguya F. Factors associated with default from treatment among tuberculosis patients in nairobi province, Kenya: A case control study. BMC Public Health 2011;11. https://doi.org/10.1186/1471-2458-11-696.

[54] Shargie EB, Lindtjørn B. Determinants of treatment adherence among smear-positive pulmonary tuberculosis patients in Southern Ethiopia. PLoS Medicine 2007;4. https://doi.org/10.1371/journal.pmed.0040037.

[55] Amuha MG, Kutyabami P, Kitutu FE, Odoi-Adome R, Kalyango JN. Non-adherence to anti-TB drugs among TB/HIV co-infected patients in Mbarara Hospital Uganda: prevalence and associated factors. Afr Health Sci 2009;9 Suppl 1.

[56] Husain MO, Dearman SP, Chaudhry IB, Rizvi N, Waheed W. Clinical Practice and Epidemiology The relationship between anxiety , depression and illness perception in tberculosis patients in Pakistan. Clinical Practice and Epidemiology in Mental Health 2008;5:4.

[57] Suhadev M, Thomas BE, Raja Sakthivel M, Murugesan P, Chandrasekaran V, Charles N, et al. Alcohol use disorders (AUD) among tuberculosis patients: A study from Chennai, South India. PLoS ONE 2011;6:e19485. https://doi.org/10.1371/journal.pone.0019485.

For peer review only

[58] Thapa P, Kamath R, Shetty BK, Monteiro A, Sekaran VC. Prevalence and Associated Factors of Alcoholism among Tuberculosis Patients in Udupi Taluk, Karnataka, India: A Cross Sectional Study. J Nepal Health Res Counc 2014;12:177–81.

[59] Peltzer K, Louw J, Mchunu G, Naidoo P, Matseke G, Tutshana B. Hazardous and harmful alcohol use and associated factors in tuberculosis public primary care patients in South Africa. International Journal of Environmental Research and Public Health 2012;9:3245–57. https://doi.org/10.3390/ijerph9093245.

[60] Necho M, Tsehay M, Seid M, Zenebe Y, Belete A, Gelaye H, et al. Prevalence and associated factors for alcohol use disorder among tuberculosis patients: a systematic review and meta-analysis study. Substance Abuse: Treatment, Prevention, and Policy 2021;16:2. https://doi.org/10.1186/s13011-020-00335-w.

[61] Veerakumar AM, Sahu SK, Sarkar S, Kattimani S, Govindarajan S. Alcohol use disorders among pulmonary tuberculosis patients under RNTCP in urban Pondicherry, India. Indian J Tuberc 2015;62:171–7. https://doi.org/10.1016/j.ijtb.2015.07.017.

[62] Taquet M, Geddes JR, Husain M, Luciano S, Harrison PJ. 6-month neurological and psychiatric outcomes in 236 379 survivors of COVID-19: a retrospective cohort study using electronic health records. The Lancet Psychiatry 2021;8:416–27. https://doi.org/10.1016/S2215-0366(21)00084-5.

[63] Lebina L, Dube M, Hlongwane K, Brahmbatt H, Lala SG, Reubenson G, et al. Trends in paediatric tuberculosis diagnoses in two south african hospitals early in the covid-19 pandemic. South African Medical Journal 2020;110:1149–50. https://doi.org/10.7196/SAMJ.2020.v110i12.15386.

For peer review only

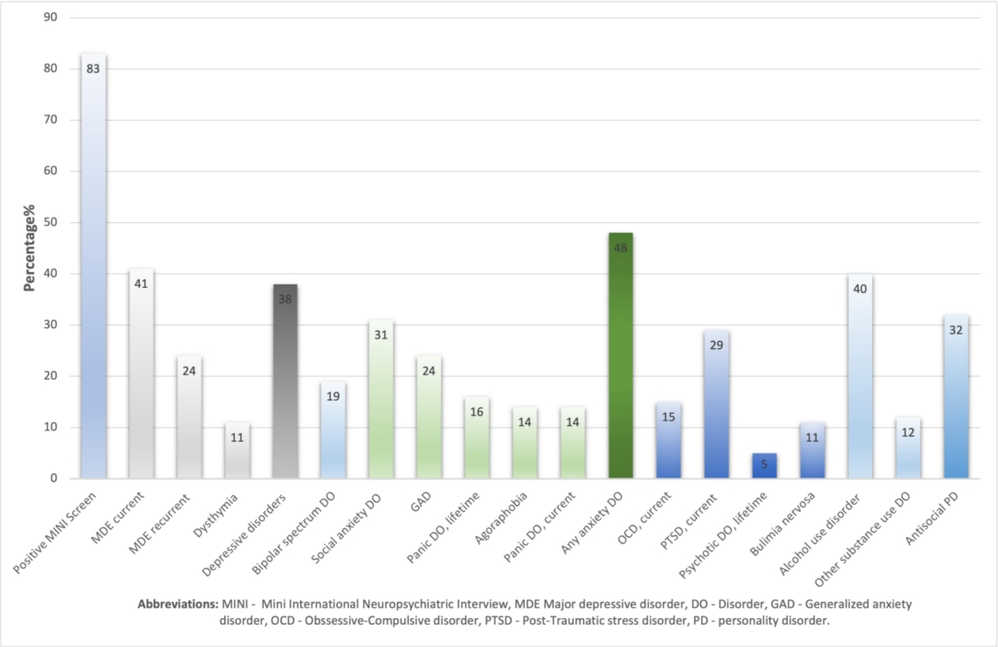

A proportion of patients with mental disorders among tuberculosis patients (N = 197)

239x155mm (330 x 330 DPI)

STROBE Statement—Checklist of items that should be included in reports of ***cross-sectional studies***

	Item No	Recommendation
**Title and abstract**	1	*(a)* Indicate the study's design with a commonly used term in the title or the abstract<br>Cross-sectional study as indicated in the Abstract on page 2 and Methods on page 5.
		(b) Provide in the abstract an informative and balanced summary of what was done and what was found<br>Provided in the Abstract on page 2.
**Introduction**		
Background/rationale	2	Explain the scientific background and rationale for the investigation being reported<br>Included in the introduction on pages 4 and 5.
Objectives	3	State-specific objectives, including any prespecified hypotheses<br>Included in the introduction on pages 4 and 5.
**Methods**		
Study design	4	Present key elements of study design early in the paper<br>Included in the Methods on page 5
Setting	5	Describe the setting, locations, and relevant dates, including periods of recruitment, exposure, follow-up, and data collection<br>Included in the Methods on pages 5, 6, and 7.
Participants	6	*(a)* Give the eligibility criteria, and the sources and methods of selection of participants<br>Included in the Methods on pages 5 and 6.
Variables	7	Clearly define all outcomes, exposures, predictors, potential confounders, and effect modifiers. Give diagnostic criteria, if applicable<br>Included in the Methods on pages 5, 6, 7, and 8.
Data sources/ measurement	8*	For each variable of interest, give sources of data and details of methods of assessment (measurement). Describe comparability of assessment methods if there is more than one group<br>Included in the Methods on pages 5, 6, 7, and 8.
Bias	9	Describe any efforts to address potential sources of bias<br>Included in the Methods on pages 5, 6, 7, and 8.
Study size	10	Explain how the study size was arrived at<br>Included in the Methods on pages 5, 6, and 7.
Quantitative variables	11	Explain how quantitative variables were handled in the analyses. If applicable, describe which groupings were chosen and why
Statistical methods	12	*(a)* Describe all statistical methods, including those used to control for confounding<br>Included in the Methods on pages 5, 6, 7, and 8.
		(b) Describe any methods used to examine subgroups and interactions<br>Included in the Methods on pages 5, 6, 7, and 8.
		(c) Explain how missing data were addressed<br>Included in the Methods on pages 5, 6, 7, and 8.

For peer review only

		(c) If applicable, describe analytical methods taking account of sampling strategy<br>Included in the Methods on pages 5, 6, 7, and 8.
		(*e*) Describe any sensitivity analyses Not applicable
**Results**		
Participants	13*	(a) Report numbers of individuals at each stage of study—eg numbers potentially eligible, examined for eligibility, confirmed eligible, included in the study, completing follow-up, and analysed<br>Included in the Results on pages 9, 10, 11, and 12
		(b) Give reasons for non-participation at each stage<br>Not applicable
		(c) Consider use of a flow diagram<br>Not required
Descriptive data	14*	(a) Give characteristics of study participants (eg demographic, clinical, social) and information on exposures and potential confounders<br>Included in the Results on page 9 and summarised in Table 1
		(b) Indicate number of participants with missing data for each variable of interest<br>Included in the Results on page 9
Outcome data	15*	Report numbers of outcome events or summary measures<br>Included in the Results on page 9, 10, 11, and 12. Also, sumamrised in figure 1
Main results	16	*(a)* Give unadjusted estimates and, if applicable, confounder-adjusted estimates and their precision (eg, 95% confidence interval). Make clear which confounders were adjusted for and why they were included<br>Included in the Results on page 9, 10, 11, and 12.
		(b) Report category boundaries when continuous variables were categorized<br>Included in the Results on page 9, 10, 11, and 12.
		(*c*) If relevant, consider translating estimates of relative risk into absolute risk for a meaningful time period – Not applicable
Other analyses	17	Report other analyses done—eg analyses of subgroups and interactions, and sensitivity analyses<br>Not applicable
**Discussion**		
Key results	18	Summarise key results with reference to study objectives<br>Included in the Discussion on pages 12 and 13
Limitations	19	Discuss limitations of the study, taking into account sources of potential bias or imprecision. Discuss both direction and magnitude of any potential bias<br>Included in the discussion on page 16
Interpretation	20	Give a cautious overall interpretation of results considering objectives, limitations, multiplicity of analyses, results from similar studies, and other relevant evidence<br>Included in the Discussion from page 12 to 16.
Generalisability	21	Discuss the generalisability (external validity) of the study results<br>Included in the Discussion on pages 16 and 17.
**Other information**		
Funding	22	Give the source of funding and the role of the funders for the present study and, if applicable, for the original study on which the present article is based<br>Provided in the text on page 17.

For peer review only - http://bmjopen.bmj.com/site/about/guidelines.xhtml

*Give information separately for exposed and unexposed groups.

Note: An Explanation and Elaboration article discusses each checklist item and gives methodological background and published examples of transparent reporting. The STROBE checklist is best used in conjunction with this article (freely available on the Web sites of PLoS Medicine at http://www.plosmedicine.org/, Annals of Internal Medicine at http://www.annals.org/, and Epidemiology at http://www.epidem.com/). Information on the STROBE Initiative is available at www.strobe-statement.org.

For peer review only
